# GATA3 Mediates a Fast, Irreversible Commitment to BMP4-Driven Differentiation in Human Embryonic Stem Cells

**DOI:** 10.1016/j.stem.2020.03.005

**Published:** 2020-05-07

**Authors:** Alexandra Gunne-Braden, Adrienne Sullivan, Borzo Gharibi, Rahuman S.M. Sheriff, Alok Maity, Yi-Fang Wang, Amelia Edwards, Ming Jiang, Michael Howell, Robert Goldstone, Roy Wollman, Philip East, Silvia D.M. Santos

**Affiliations:** 1 The Francis Crick Institute, London, UK; 2 European Molecular Biology Laboratory – European Bioinformatics Institute (EMBL-EBI), Hinxton, Cambridgeshire, UK; 3 University of California, Los Angeles (UCLA), Los Angeles, CA, USA; 4 MRC-LMS Imperial College London, London, UK

**Keywords:** hESC, differentiation, commitment, GATA3, BMP4, fate decisions, positive feedback, bistability

## Abstract

During early development, extrinsic triggers prompt pluripotent cells to begin the process of differentiation. When and how human embryonic stem cells (hESCs) irreversibly commit to differentiation is a fundamental yet unanswered question. By combining single-cell imaging, genomic approaches, and mathematical modeling, we find that hESCs commit to exiting pluripotency unexpectedly early. We show that bone morphogenetic protein 4 (BMP4), an important differentiation trigger, induces a subset of early genes to mirror the sustained, bistable dynamics of upstream signaling. Induction of one of these genes, GATA3, drives differentiation in the absence of BMP4. Conversely, GATA3 knockout delays differentiation and prevents fast commitment to differentiation. We show that positive feedback at the level of the GATA3-BMP4 axis induces fast, irreversible commitment to differentiation. We propose that early commitment may be a feature of BMP-driven fate choices and that interlinked feedback is the molecular basis for an irreversible transition from pluripotency to differentiation.

## Highlights

• Irreversible commitment to BMP4-driven hESC differentiation is fast
• SMAD activation is sustained, bistable, and irreversible due to positive feedback
• GATA3 mirrors SMAD dynamics and mediates fast commitment to differentiation
• GATA3 is an early commitment gene

## Graphical Abstract

**Figure:**
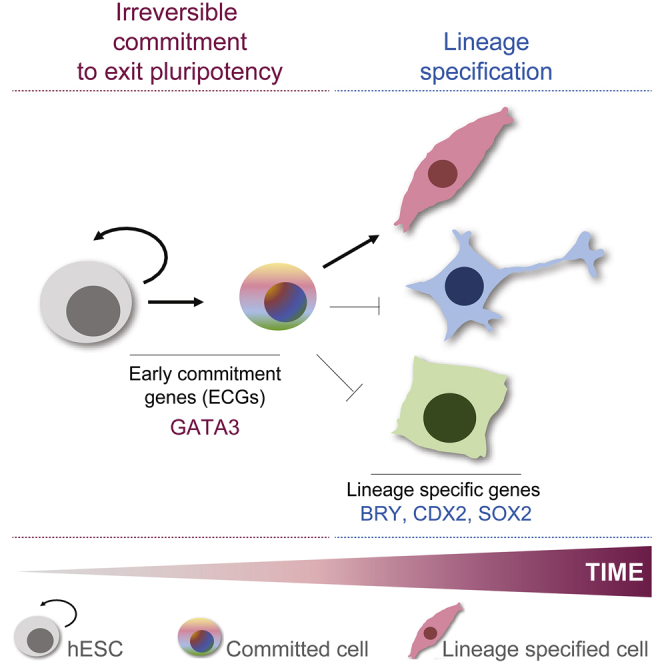

## Teaser

Gunne-Braden et al. show that GATA3 is an early commitment gene that mediates fast commitment to BMP4-driven differentiation in human embryonic stem cells (hESCs). Interlinked feedback regulation at the level of the GATA3-BMP4 axis allows for an irreversible exit from pluripotency and an early commitment to differentiation.

## Introduction

During early development, extrinsic triggers prompt a collection of pluripotent cells in the blastocyst to begin the dramatic and long process of differentiation that gives rise to the tissues of the three germ layers (endoderm, mesoderm, and ectoderm). Precise temporal control during these early fate choices is paramount and affects the success of differentiation (Kojima et al., 2014). These early cellular decisions are, however, still poorly characterized. In particular, when and how embryonic cells irreversibly lose the ability to maintain the pluripotent state (i.e., irreversibly commit to differentiate) is a fundamental, yet unanswered question. Poised to differentiate, human embryonic stem cells (hESCs) are an invaluable model to address this question.

Bone morphogenetic protein 4 (BMP4) is an important instructive cue known to drive differentiation during early development (Dale et al., 1992). BMP4 has been shown to be essential for embryogenesis, predominantly for mesoderm and cardiac formation (Dale et al., 1992). BMP4 knockout (KO) mice are embryonic lethal and lack mesoderm differentiation (Winnier et al., 1995), highlighting its essential role in early gastrulation. Elegant work has shown that the stimulation of hESCs in 2D confinement recapitulates early embryonic germ layer formation, and, in particular, mesendoderm formation (Warmflash et al., 2014; Etoc et al., 2016). These observations suggest that BMP4 is a trigger for pluripotency exit and early cellular differentiation.

During BMP4-driven differentiation of hESCs, a precise coordination of sequential hallmark events occurs: cells change their morphology and motility by elongating in size and quickly moving away from the colony; there is extensive remodeling of gene expression programs, whereby pluripotent, stem cell-associated markers are downregulated and lineage-specific markers are upregulated; a plethora of epigenetic changes take place to alter acetylation and methylation profiles and remodel chromatin states; and cell division cycles lengthen (Boheler, 2009, Xie et al., 2013, Calder et al., 2013, Gifford et al., 2013). The onset of these hallmark changes is believed to be the point of no return toward differentiation and lineage specification. However, all of these changes are relatively late events during differentiation, with most occurring 48 to 72 h after cells are first exposed to BMP4 cues. The question is thus: in this precise sequence of events, when does irreversible commitment to exit pluripotency and undergo differentiation take place?

## Results

### BMP4 Drives Fast Commitment to Differentiation in hESCs

To understand when hESCs first commit to differentiation, we sought to determine the minimum exposure to differentiation cues that elicit changes in morphology and gene expression, characteristic of both fate specification and pluripotency loss.

BMP4 stimulation of hESCs in the presence of basic fetal growth factor 2 (FGF2) is thought to mediate cardiac (lateral) mesoderm differentiation (Yu et al., 2011), as seen by the upregulation of canonical mesoderm genes such as BRACHYURY (BRY), CDX2, MIXL1, HAND1, TBX3, and GATA4, 72 h after BMP4 treatment (Figure S1A).

**Figure 1:**
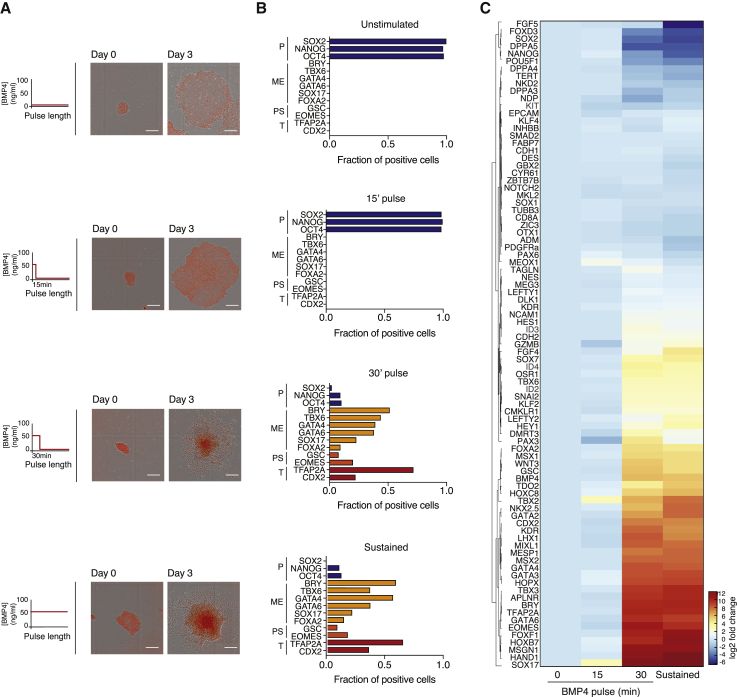
Irreversible Commitment Differentiation Is an Early Event in hESCs (A) Left: schematic of duration of BMP4 pulses used to drive hESC differentiation. Right: representative images of hESC colonies tagged with NLS-mCherry at days 0 and 3 of BMP4 (50 ng/mL) treatment. Scale bar represents 100 μm. (B) Quantification of the fraction of positive cells showing expression of 13 pluripotency (P), mesendoderm (ME), primitive streak (PS), and trophoblast (T) markers at the protein level at day 3 following different pulses of BMP4 stimulation. (C) Heatmap comparing the expression level of 88 genes involved in pluripotency and differentiation after different pulses of BMP4 stimulation and analyzed by qPCR. The housekeeping gene GUSB was used for normalization. n = 2 independent experiments.

To test the minimum duration of BMP4 stimulation required for cells to express these markers, hESCs were challenged with BMP4 pulses of different lengths, ranging from a short pulse of 15 min to a sustained stimulation of cells for 72 h (Figure 1A), followed by washing and culturing cells in hESCs back in pluripotency growth media (mTeSR). Sustained BMP4 stimulation resulted in the expected global changes in cell morphology, as well as in the downregulation of the stem cell genes NANOG, SOX2, and OCT4, and the upregulation of mesendoderm and primitive streak-specific markers, including BRY, GSC, CDX2, TBX6, and GATA4 (Figures 1A and 1B). Stimulating cells with a short 15 min pulse of BMP4 demonstrated little effect on cell morphology and cells continued to rapidly proliferate and maintain the characteristic gene expression signature of untreated, pluripotent cells (Figures 1A and 1B). Unexpectedly, a slightly longer, 30 min pulse of BMP4 triggered 88% of the cells to undergo the same dramatic morphological changes, loss of pluripotency-specific genes, and upregulation of differentiation markers as cells that had been treated with sustained BMP4 for 72 h (Figures 1A and 1B). This was further confirmed by monitoring the gene expression of 90 different pluripotency and lineage-specifying genes (Figure 1C), highlighting the similarity in gene expression signature between a pulse of 30 min and a sustained 72 h BMP4 stimulation. Similar results were also seen for different hESC lines (Figures S1B–S1E) and when using three alternative mesoderm differentiation protocols (Figure S1F), suggesting that this is a conserved feature of BMP4-driven differentiation of hESCs. These observations suggest that while known hallmark events in differentiation happen days after cells are exposed to BMP-driven differentiation cues, the irreversible commitment to leave pluripotency and undergo differentiation is remarkably fast.

### Switch-like, Irreversible Activation of the SMAD Regulatory Network Sustains BMP4 Signals

Given that hESCs commit to differentiation so soon after being exposed to pro-differentiation cues, we reasoned that commitment may be encoded by and depend on how BMP4 signals are first interpreted: at the level of signal transduction networks and the resulting first waves of gene expression.

**Figure 2:**
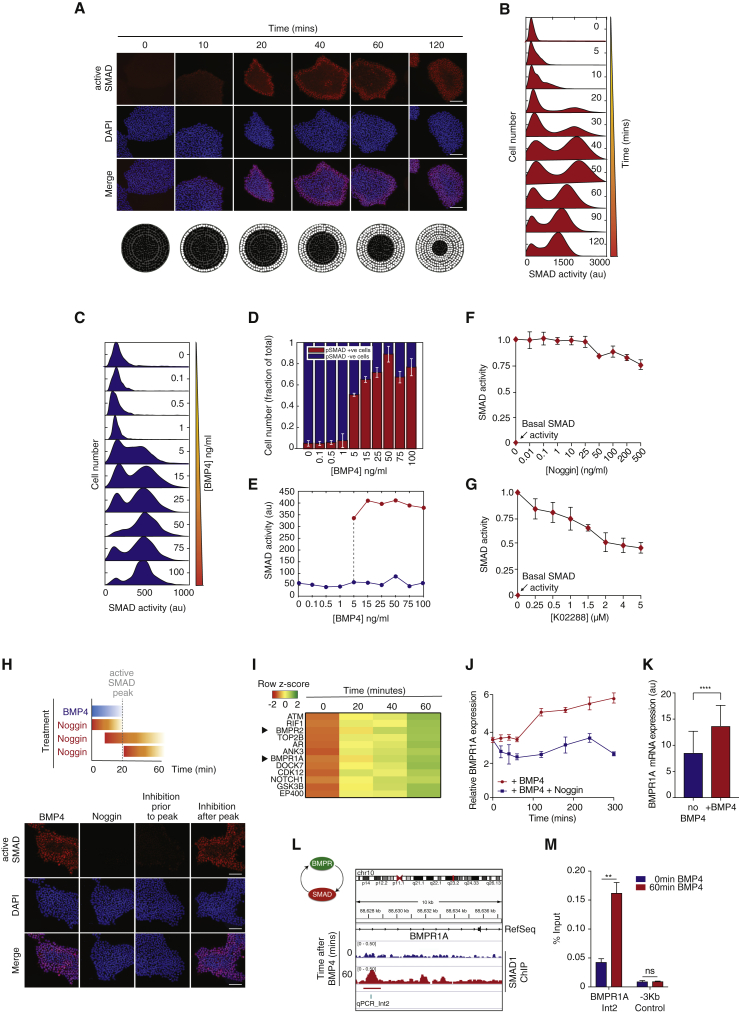
Positive Feedback Promotes Switch-like, Irreversible Activation of the SMAD Regulatory Network and Sustains BMP4 Signals (A) Top: representative images of SMAD activation dynamics after BMP4 (50 ng/mL) treatment. Scale bar represents 100 μm. Bottom: SMAD activation dynamics in space after BMP4 treatment, assuming a circular geometry for hESC colonies. (B) Quantification of SMAD activation after BMP4 (50 ng/mL) treatment. More than 200 cells were analyzed for each experimental condition. n = 3 independent experiments. (C) Quantification of SMAD activation dynamics as a function of BMP4 concentration. (D) Percentage of cells showing active (red) and inactive (blue) SMAD as a function of BMP4 concentration. Error bars represent means ± standard deviations (SDs). (E) Mean intensity of fitted Gaussian distributions corresponding to cells with active (red) and inactive (blue) SMAD for a range of BMP4 concentrations. The dotted line highlights the BMP4 concentration at which two populations of active and inactive SMAD are seen. More than 200 cells were analyzed for each experimental condition. n = 3 independent experiments. (F) Quantification of SMAD activity at steady state (60 min) in response to increasing concentrations of Noggin. Peak levels of active SMAD were normalized to 1. Basal activity at time 0 is shown. n > 200 cells were analyzed for each experimental condition. n = 3 independent experiments. Error bars represent means ± standard deviations (SDs). (G) Quantification of SMAD activity at steady state (60 min) in response to increasing concentrations of BMP receptor inhibitor (K02288). Peak levels of active SMAD were normalized to 1. Basal activity at time 0 is shown. n > 200 cells were analyzed for each experimental condition. n = 3 independent experiments. Error bars represent means ± standard deviations (SDs) (H) Top: schematic showing time of BMP4 and Noggin stimulation for each experimental condition. Bottom: representative images of SMAD activation after BMP4 stimulation followed by Noggin (100 ng/mL) treatment before or after SMAD full activation. Cells cultured with either BMP4 or Noggin alone were used as positive and negative controls, respectively. Scale bars represent 100 μm. n > 200 cells were analyzed for each experimental condition. (I) Heatmap showing a subset of RNA-seq-based gene expression profiles showing graded expression dynamics after BMP4 stimulation. BMP type I and type II receptors are highlighted. (J) Quantification of BMP receptor (BMPR1A) expression after BMP4 stimulation in the presence (blue) or absence (red) of Noggin (100 ng/mL) as measured by qPCR. Housekeeping gene GUSB was used for normalization. Error bars represent ± SDs. n = 3 independent experiments. (K) Quantification of BMP receptor (BMPR1A) expression in the presence or absence of BMP4 (50 ng/mL), as measured by mRNA-FISH. Error bars represent ±SDs. n > 200 cells were analyzed for each experimental condition (∗∗∗∗p < 0.01). (L) SMAD1 ChIP-seq analysis of intron 2 of BMPR1A in the presence (red) or absence (blue) of BMP4 showing positive feedback between SMAD and its activator, BMPR1A. Significant peak region relative to input chromatin is highlighted. The amplicon used for ChIP qPCR (qPCR_Int2) is shown. (M) Quantification of SMAD1 ChIP-qPCR using qPCR_Int2 as an amplicon in the presence (red) or absence (blue) of BMP4 (50 ng/mL). A primer set −3 kb from BMPR1A was used as a negative control. Error bars represent means ± SDs for n = 3 biological replicates (^∗∗^p < 0.01, ns is not significant).

BMP4 binds to BMP receptors at the plasma membrane, and the ensuing signals are transduced by the canonical SMAD transduction network, the first interpreters of BMP4 signals (Liu et al., 1996, Massagué et al., 2005). In hESCs, we see that BMP4 stimulation results in a sustained SMAD1/5/8 activation for at least 2 h, as measured by SMAD1/5/8 phosphorylation and their nuclear translocation (Figures 2A and 2B). SMAD1/5/8 are first activated in cells at the periphery of hESC colonies, and with time, an increasing number of cells become activated toward the colony center in a switch-like fashion (Figure 2A). In fact, this pattern of SMAD activation appears to mirror the self-organization of lineage-specific markers known to occur in hESCs (Warmflash et al., 2014).

The bimodal, switch-like SMAD1/5/8 dynamics were also recapitulated when hESCs were challenged for 1 h with increasing doses of BMP4 (Figures 2C–2E). At sub-threshold concentrations of BMP4, the pathway is inactive. After this threshold has been met, for the same BMP4 concentration, two different populations of cells with either active or inactive SMAD1/5/8 are seen for a single concentration of BMP4. This is characteristic of switch-like, bistable systems (Figures 2C–2E).

Bistability is a recurrent theme in cellular transitions and can give rise to irreversible responses (Pomerening et al., 2003, Santos et al., 2007). Bistability in the SMAD1/5/8 network could promote a short-term dynamically maintained memory of the BMP4 stimulus. Hysteresis describes a state whereby the amount of stimulus to maintain a system as active is much smaller than the amount of stimulus needed to activate the system. Hysteresis to the point of irreversibility is therefore a hallmark of bistability. We tested how a decrease in stimulus (either by removal of BMP4 or by adding pathway inhibitors) after full SMAD1/5/8 activation affected their activation dynamics (Figure S2A). After full activation of SMAD1/5/8, both the complete washout of BMP4 (Figures S2B and S2C) or treatment with the BMP4 inhibitor Noggin (a BMP4 mimetic) proved unable to significantly reduce SMAD1/5/8 activity (Figures 2F and S2C). To exclude the possibility that a small fraction of internalized BMP4 receptor complexes were still signaling to activate SMAD1/5/8, a specific BMP receptor inhibitor was used to block the pathway. As expected for hysteretic responses, once the pathway was fully activated, a fraction (∼50%) of SMAD1/5/8 activity was still maintained after BMP receptor inactivation (Figures 2G and S2C).

To understand whether the irreversibility in SMAD response depends on a specific BMP4-driven SMAD activation threshold, Noggin, which binds and inhibits BMP4, was added to hESCs either before or after full SMAD activation (Figure 2H). When Noggin was added after full SMAD activation, it had little effect on inhibiting SMAD activity, while SMAD1/5/8 dynamics were no longer sustained when cells were treated with Noggin before full SMAD activation (Figure 2H). This indicates that a threshold of SMAD activation must be met for irreversibility. We next tested whether there was a fixed, absolute BMP4 concentration threshold required for full SMAD1/5/8 activation by stimulating cells with increasing doses of BMP4 for different lengths of time (Figure S2D). We saw that the appearance of an active SMAD1/5/8 population was dependent on both the strength and the duration of BMP4 signals (Figure S2D). In other words, hESCs that were stimulated for longer periods needed less BMP4 to activate SMAD compared to cells that were exposed to a shorter pulse of BMP4. These data suggest that upon BMP4 stimulation, SMAD activation is bistable and that the SMAD pathway integrates both the amplitude and duration of BMP4 cues.

### Positive Feedback Sustains SMAD Activity

To explore how switch-like, sustained SMAD activation dynamics are brought about, hESCs stimulated with BMP4 were challenged with the commonly used transcription and translation inhibitors actinomycin D and cycloheximide, respectively (Figures S2E–S2G). Blocking translation resulted in a transient activation of SMAD1/5/8, whereas blocking transcription resulted in a remarkable decrease in both the amplitude and duration of SMAD1/5/8 dynamics (Figures S2E–S2G). This suggests that early gene expression and subsequent protein translation are at the heart of maintaining SMAD activation dynamics after BMP4 cues.

To achieve a comprehensive understanding of early gene expression at the very onset of BMP4-driven differentiation, transcriptome analysis (RNA sequencing [RNA-seq]) of BMP4-treated hESCs was performed (Figure S2H). Correlation analysis of the RNA-seq data identified four major clusters of differentially expressed genes, based on whether their expression was up- or downregulated after BMP4 stimulation and whether the expression profiles showed either graded or switch-like dynamics (Figure S2H). Notably, a cluster containing 488 differentially expressed genes highlighted both BMP type I and type II receptors as being upregulated in a graded manner after BMP4 stimulation (Figures 2I and S2I). In particular, the upregulation of BMP type I receptor (BMPR1A), the main activator of SMAD1/5/8 (Shi and Massagué, 2003), suggests that after BMP4 stimulation, SMAD activates its own activator, which is a classical positive feedback loop that could sustain its activity.

The upregulation of BMPR1A after BMP4 treatment was further confirmed by qPCR (Figure 2J) and single-molecule RNA fluorescence *in situ* hybridization (RNA-FISH) (Figures 2K and S2J). Chromatin immunoprecipitation sequencing (ChIP-seq) experiments identified specific SMAD sites within an intron of BMPR1A, confirming that BMPR1A expression is likely to depend specifically on SMAD1/5/8 and on BMP4 stimulation (Figures 2L, 2M, and S2K). This suggests that positive feedback regulation underlies the switch-like SMAD activation dynamics to BMP4 signals.

### GATA3 Mirrors SMAD-like, Irreversible Activation Dynamics and Decodes BMP4 Signals

**Figure 3:**
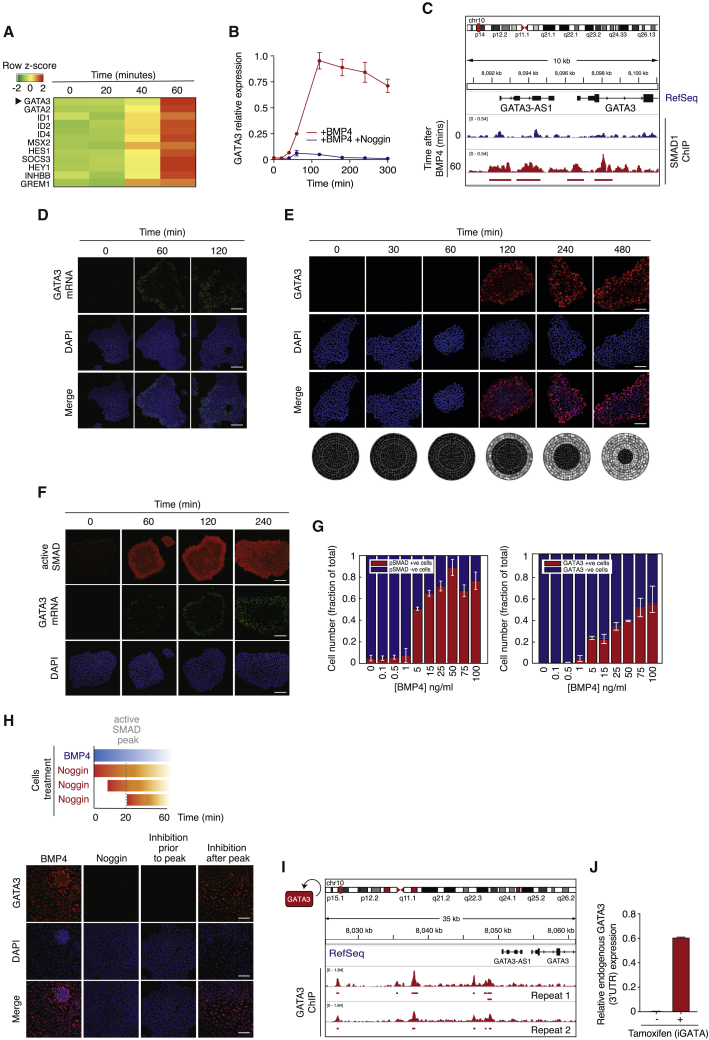
GATA3 Mirrors SMAD Switch-like, Irreversible Activation Dynamics and Decodes BMP4 Signals (A) Heatmap of a subset of RNA-seq-based gene expression profiles showing switch-like dynamics for differentially expressed genes after BMP4 stimulation. The GATA3 gene is highlighted. (B) Quantification of GATA3 expression after BMP4 stimulation in the presence (blue) or absence (red) of Noggin (100 ng/mL) as measured by qPCR. The housekeeping gene GUSB was used for normalization. Error bars represent ±SDs from n = 3 biological replicates. (C) SMAD1 ChIP-seq analysis of the early promoter region of GATA3 in the presence (red) or absence (blue) of BMP4. Significant peak regions relative to input chromatin are highlighted. Error bars represent means ± standard deviations (SDs) (D) Representative images of GATA3 mRNA levels after BMP4 (50 ng/mL) treatment as measured by mRNA-FISH. Scale bar represents 100 μm. (E) Top: representative images of GATA3 protein expression after BMP4 (50 ng/mL) treatment. Scale bar represents 100 μm. Bottom: GATA3 expression in space after BMP4 treatment, assuming a circular geometry for hESC colonies. (F) Representative images of SMAD activation and GATA3 mRNA expression in single cells after BMP4 (50 ng/mL) treatment. Scale bar represents 100 μm. (G) Quantification of the steady-state fraction of SMAD and GATA3 positive (red) and negative (blue) cells as a function of BMP4 concentration. Error bars represent means ± SDs. (H) Top: schematic showing time of BMP4 and Noggin stimulation for each experimental condition. Bottom: representative images of GATA3 expression after BMP4 stimulation followed by Noggin (100 ng/mL) treatment before or after SMAD full activation. Cells cultured with either BMP4 or Noggin alone were used as positive and negative controls, respectively. Scale bars represent 100 μm. n > 200 cells were analyzed for each experimental condition. (I) GATA3 ChIP-seq analysis of its own promoter after BMP4 stimulation showing potential autoregulation. Significant peak regions relative to input chromatin are highlighted. n = 2 biological replicates are shown. (J) Endogenous GATA3 mRNA expression levels after GATA3 induction by tamoxifen in iGATA3-expressing hESCs, as measured by qPCR. The housekeeping gene GUSB was used for normalization. Error bars represent ±SDs from n = 3 biological replicates.

We next investigated how SMAD dynamics may be decoded to give rise to the observed fast, irreversible commitment to undergo BMP-driven differentiation. The RNA-seq analysis also highlighted a cluster of 138 genes implicated in developmental processes and differentiation (Figure S2H). Many of the genes within this cluster are known canonical SMAD signaling targets (including ID1, ID2, and ID4) and all were upregulated in a switch-like manner after BMP4 stimulation (Figures 3A, S3A, and S3B). The most significant differentially expressed gene was GATA3, a gene first identified in T cell development that belongs to the GATA family of transcription factors (Oosterwegel et al., 1992). GATA3 has a known role in early development during trophectoderm specification (Home et al., 2009, Blakeley et al., 2015, Krendl et al., 2017), but it has not been associated with SMAD signaling in hESCs. However, we find that the transcriptional regulation of GATA3 is likely to be directly controlled by SMAD, as ChIP-seq and ChIP-qPCR analyses showed extensive SMAD1/5/8 binding in the early promoter region of GATA3 in response to BMP4 (Figures 3B, 3C, S3C, and S3D).

After BMP4 stimulation, GATA3 expression is sustained at both the mRNA (Figure 3D) and protein levels for at least 8 h (Figure 3E). GATA3 expression displays a spatial switch-like pattern of expression, whereby cells at the periphery of the colony express GATA3 first, and with time, an increasing number of cells become GATA3^+^ (Figure 3E). This is reminiscent of the upstream SMAD1/5/8 activation dynamics, and GATA3 expression in single cells closely mirrored that of SMAD activation, both as a function of time (Figure 3F) and BMP4 concentration (Figure 3G). In other words, when (and where) SMAD1/5/8 is active within the hESC colony, GATA3 is expressed.

We therefore postulated that a switch-like activation of SMAD1/5/8 could result in a switch-like, irreversible expression of GATA3. To investigate this, hESCs stimulated with BMP4 were treated with the BMP4 inhibitor Noggin either before or right after reaching a peak of SMAD activation (Figure 3H). Treatment with Noggin before the full activation of SMAD resulted in the absence of GATA3 expression. However, once a threshold of SMAD activation was reached, and even in the absence of BMP4 signals, GATA3 expression was maintained (Figure 3H), strongly supporting irreversibility in GATA3 expression. These observations suggest that the switch-like activation of SMAD1/5/8 results in the switch-like, irreversible expression of a subset of downstream SMAD-specific targets such as GATA3.

### Positive Feedback Sustains GATA3 Expression and Sustains BMP4 Signals

We thus wondered whether positive feedback could also be the molecular mechanism underlying the irreversibility in GATA3 expression after BMP4 stimulation. To that effect, ChIP-seq and ChIP-qPCR analyses of GATA3 after BMP4 stimulation showed that GATA3 expression could be autoregulatory (Figures 3I, S3E, and S3F), as described for T cells (Oosterwegel et al., 1992, Höfer et al., 2002; Ouyang et al., 2000). To test this further, we established a stable hESC line expressing a tamoxifen-responsive inducible GATA3. In the absence of any BMP4 cues, the induction of GATA3 nuclear translocation in pluripotent hESCs with tamoxifen for 96 h led to a remarkable induction of endogenous GATA3 mRNA (Figure 3J), confirming that in hESCs, GATA3 expression is also autoregulatory.

**Figure 4:**
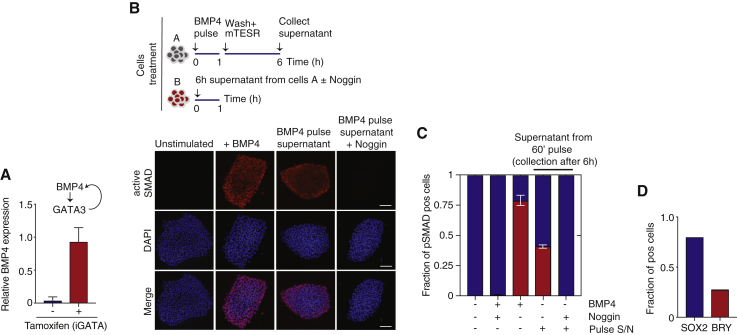
Positive Feedback from GATA3 to BMP4 Mediates Propagation of Commitment to Differentiation throughout hESC Colonies (A) Endogenous BMP4 mRNA expression levels after GATA3 induction by tamoxifen in iGATA3-expressing hESCs, as measured by qPCR. The housekeeping gene GUS was used for normalization. Error bars represent ±SDs from n = 2 biological replicates. (B) Top: schematic showing treatments for hESCs A and B to test whether diffusible factors are responsible for early commitment to differentiation after a pulse of BMP4 stimulation. Bottom: representative images of SMAD activation in hESCs B after following 1 h treatment with conditioned media from cells subjected to a BMP4 pulse (6 h supernatant). Cells cultured with either BMP4 (1 h) in the presence or absence of Noggin or left untreated were used as positive and negative controls, respectively. Scale bars represent 100 μm. (C) Quantification of the fraction of positive B cells for active SMAD (pSMAD) after the different treatments. n = 2 independent experiments. (D) Quantification of the fraction of positive B cells for BRACHYURY (BRY) and SOX2 genes. More than 200 cells were analyzed. n = 2 independent experiments.

We also found that GATA3 regulates the expression of the BMP4 gene (Figures S4A and S4B) and that the dynamic induction of GATA3 activity in hESCs results in the upregulation of BMP4 in the absence of exogenous BMP4 signals (Figure 4A). These observations support the finding that GATA3 expression is embedded in positive feedback regulation that ensures maintenance of the expression of GATA3 and continued activation of upstream signaling.

### GATA3-Induced BMP4 Underlies Continued Commitment to Differentiation

Positive feedback regulation by GATA3-driven BMP4 expression could explain why a short pulse of BMP4 results in a long-term commitment to differentiation in the entire hESC colony. In other words, a positive feedback loop inducing BMP4 in neighboring cells could explain why cells that may not have experienced SMAD activation and hence do not express GATA3 still undergo differentiation.

We tested the hypothesis of whether diffusible factors (e.g., BMP4) could result in signal propagation and early commitment to differentiation after a short pulse of BMP4 (Figure 4B). To this end, a population of hESCs (pop A) were stimulated for 1 h with BMP4, after which BMP4 was washed away and cells maintained in mTeSR. After 6 h, the supernatant of pop A was collected and used to treat naive cells (pop B), which had not been exposed to BMP4 (Figure 4A). We observed that a 1-h treatment of pop B with the conditioned media from pop A resulted in the activation of SMAD signaling in pop B (Figures 4B and 4C). SMAD activation was abrogated if the BMP4 inhibitor Noggin was added to the conditioned media, showing that diffusible BMP4 cues are likely to be the main factor secreted by BMP4-treated cells (Figures 4B and 4C). In addition, treating pop B for 48 h with pop A conditioned media resulted in the activation of BRY expression, a canonical mesoderm differentiation marker (Figure 4D). These results suggest that conditioned media from a short pulse of BMP4 contains diffusible BMP4 cues that allow for sustained signal propagation and commitment to differentiation.

### Interlinked Positive Feedback Mediates Long-Term Memory of SMAD and GATA3 Activation

Irreversible commitment to fate choice can in principle be mediated by a single positive feedback loop (Xiong and Ferrell, 2003). We therefore built a set of ordinary differential equation-based (ODE) models to assess the contribution of the measured sequential feedback loops in the context of BMP-driven hESC differentiation.

Interlinked feedback control often underlies irreversible fate decisions (Brandman et al., 2005, Ferrell, 2013). As such, we constructed models of three simple topologies: (1) linear, (2) single positive feedback loop, and (3) interlinked positive feedback loops (Figure S4C; Supplemental Information). Stability analysis of the three models showed that the single SMAD → BMPR → SMAD feedback gave rise to a sharp, sigmoidal activation of SMAD, and consequently, to the switch-like expression of GATA3 (Figure S4C). This feedback alone, however, was insufficient to drive irreversibility. However, the model predicted that the combination of the measured SMAD- and GATA3-mediated loops could together induce, at a wide range of parameters, a hysteretic, S-shaped response to BMP4 signals, characteristic of irreversible systems (Figure S4C). This suggests that interlinked feedback control could bring about the establishment of a long-term cellular memory of SMAD and GATA3 activation and potentially underlie an irreversible commitment to cellular differentiation.

### GATA3 Is Crucial for Fast, Timely Differentiation Triggered by BMP4

**Figure 5:**
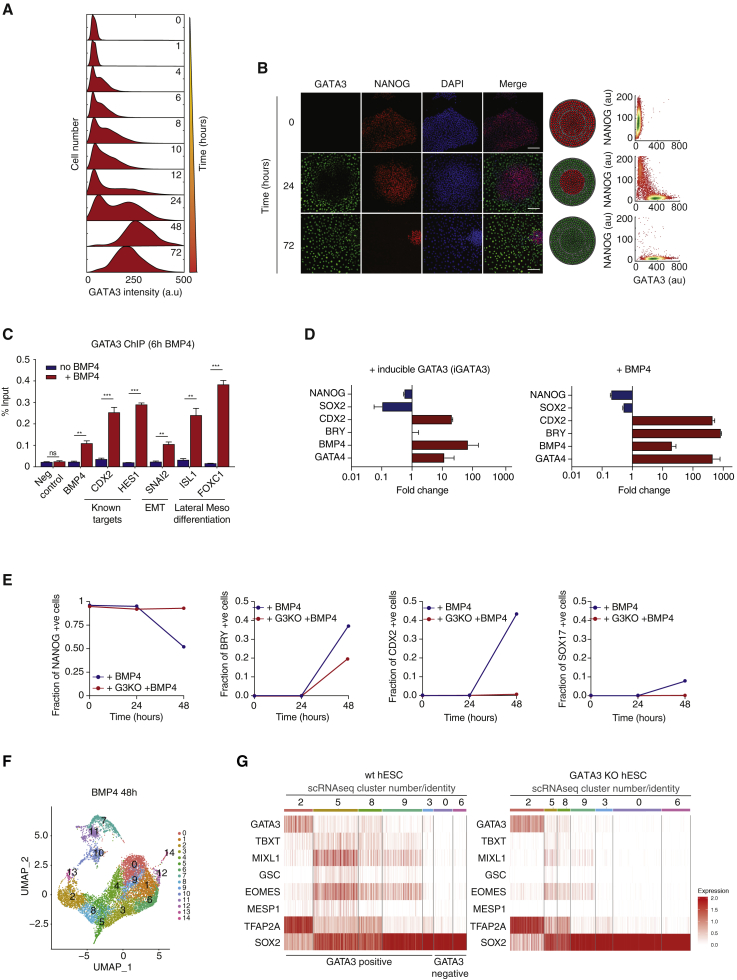
GATA3 Is Necessary for Timely Differentiation and Can Drive Differentiation in the Absence of BMP4 Signals (A) Quantification of GATA3 expression during 3 days of BMP4 (50 ng/mL) treatment. (B) Left: representative images of GATA3 and NANOG expression after BMP4 (50 ng/mL) treatment. Scale bar represents 100 μm. Center: GATA3 and NANOG expression in space after BMP4 stimulation, assuming a spherical geometry for hESC colonies. Right: quantification of GATA3 and NANOG levels in single cells after BMP4 treatment. More than 10,000 cells were analyzed for each experimental condition. n = 3 independent experiments. (C) Quantification of GATA3 ChIP-qPCR in the presence (red) or absence (blue) of BMP4 (50 ng/mL) treatment of hESC (for 6 h). BMP4, HES1, CDX2, and SNAI2 are genes shown to be involved in hESC differentiation. ISL1 and FOXC1 are lateral mesoderm (cardiac)-specific genes. A primer set −3 kb from BMP4 was used as a negative control (Neg control). Error bars represent means ± SDs for n = 2 biological replicates (^∗∗^p < 0.001, ^∗∗∗^p < 0.0001, ns is not significant). (D) Quantification of fold change of pluripotency (blue) and lineage-specific (red) genes in response to BMP4 stimulation (left) or tamoxifen-induced GATA3 expression (iGATA3) (right) relative to untreated control cells. The housekeeping gene GUSB was used for normalization. Error bars represent ±SDs from n = 3 biological replicates. (E) Quantification of the fraction of positive cells for pluripotency gene NANOG and lineage-specific markers BRY, SOX17, and CDX2 in response to BMP4 (50 ng/mL) stimulation in GATA3^−/−^ (GATA3 KO) cells (red) or control cells (blue). n > 500 cells were analyzed for each experimental condition. (F) scRNA-seq data of wild type (WT) and GATA3 KO hESC stimulated with BMP4 for 48 h. Combined cell transcriptomes were analyzed with a mean gene per cell value of ∼6,500 and a total read depth of ∼600 million reads per cell. Cell transcriptomes were projected onto a diffusion map with shared 14 nearest-neighbor clusters highlighted (first 30 dimensions, resolution 0.8). (G) Heatmaps showing normalized counts per cell for GATA3 and mesoderm differentiation genes for WT (left) and GATA3 KO (right) hESCs after 48 h BMP4 stimulation. The left panel highlights a gradient of GATA3 expression.

Next, we sought to understand the implications of these findings to the early commitment to differentiation. Previous studies have suggested that GATA3 is a lineage-specific marker in trophoblast differentiation (Home et al., 2009, Blakeley et al., 2015, Krendl et al., 2017). However, in hESCs, GATA3 protein is continually induced throughout BMP4-driven differentiation (Figures 5A and S7C–S7E). In addition, at 48–72 h post-stimulation, GATA3 co-expresses with canonical early mesoderm markers (e.g., BRY, GSC, MIXL1) (Figure 5D). This led us to hypothesize that GATA3 may have different early functions downstream of a BMP4 trigger and may act as an early commitment gene in this context, expressed days before the classical lineage-specification genes are switched on and before other known early hallmark events in BMP-driven differentiation take place (e.g., changes in morphology). As such, GATA3 could provide a link between SMAD dynamics and commitment to differentiation.

If GATA3 is an early commitment gene mediating BMP4 differentiation, then the expectation is that it should fulfill the following criteria: (1) be expressed early during pluripotency exit; (2) when expressed in hESCs, it should induce differentiation; and (3) when absent, hESC differentiation should be perturbed.

As shown before, GATA3 is expressed at the mRNA level for 40–60 min (Figures 3A, 3B, 3D, and S3A) and at the protein level 2 h after BMP4 stimulation (Figures 3E and 5A). In addition, GATA3 spatiotemporal expression shows an inverse relation with that of NANOG, a well-established pluripotency factor (Chambers et al., 2003) (Figure 5B). In line with potential early functions of GATA3, ChIP-qPCR analysis shows that in the first hours of BMP4 stimulation, GATA3 is bound to the promoters and potentially regulates canonical differentiation-associated genes, including HES1, SNAI2, ISL1, FOXC1, and CDX2 (Figure 5C). In addition, GATA3 has been shown to interact with a variety of chromatin remodeling, cellular differentiation, and cellular morphogenesis factors (Figure S5A), implying that GATA3 may act early to help mediate sequential events during differentiation (i.e., morphological changes, chromatin remodeling, upregulation of lineage specific genes, and downregulation of stem cell factors).

We reasoned that one way to explore whether GATA3 acts an early commitment marker downstream of BMP signaling was to create a synthetic system in which GATA3 expression could be induced dynamically in pluripotent hESCs, independently of BMP4 cues (Figures S5B–S5D). This strategy has been successfully used before to induce GATA1 (Ezoe et al., 2005).

We observed that in the absence of BMP4, the induction of GATA3 nuclear expression resulted in the downregulation of pluripotency genes and the upregulation of canonical differentiation genes (Figure 5C). While there were expected differences in the amplitude and expression profiles of differentiation genes compared to BMP4 stimulation, as BMP4 is expected to activate a plethora of signals (for example, the expression of BRY is known to depend on both BMP4 and on Wnt signaling, [Yamaguchi et al., 1999, Lindsley et al., 2006]), GATA3 alone was capable of driving differentiation (Figure 5D).

Furthermore, we note that perturbing GATA3 expression in hESCs, either by deleting GATA3 using a CRISPR-mediated GATA3 knockout (Figures S5F–S5I) or by downregulating GATA3 with RNA interference (Figures S5J and S5K), delays differentiation after BMP4 (Figures 5E and S5L). When GATA3 expression was compromised, the upregulation of differentiation genes (e.g., BRY, CDX2, SOX17) was profoundly delayed after BMP4 stimulation (Figures 5E and S5L).

In addition to mesoderm genes, the expression of early-gastrulation genes (MIXL1, EOMES, GSC) was compromised in GATA3 KO cells at the single-cell level, as measured by single-cell RNA-seq (scRNA-seq) (Figures 5F and 5G). The shifts in cluster sizes reflect the number of cells within each cluster and show that GATA3 KO has an effect on the number of cells expressing the highlighted genes. GATA3 KO increases the pluripotent population (clusters 3, 0, and 6) and decreases the differentiated population of cells (clusters 5, 8, and 9). In other words, GATA3 KO delays BMP4-driven differentiation. These results suggest that GATA3 is necessary for timely BMP4-driven differentiation.

### GATA3 Is Required for the Fast Commitment to BMP4-Driven Differentiation

If the hypothesis that GATA3 drives an early and irreversible differentiation response to BMP4 is correct, then the expectation would be that hESCs in which GATA3 has been knocked out or knocked down (KD) would fail to commit to differentiation following a short pulse of BMP4.

**Figure 6:**
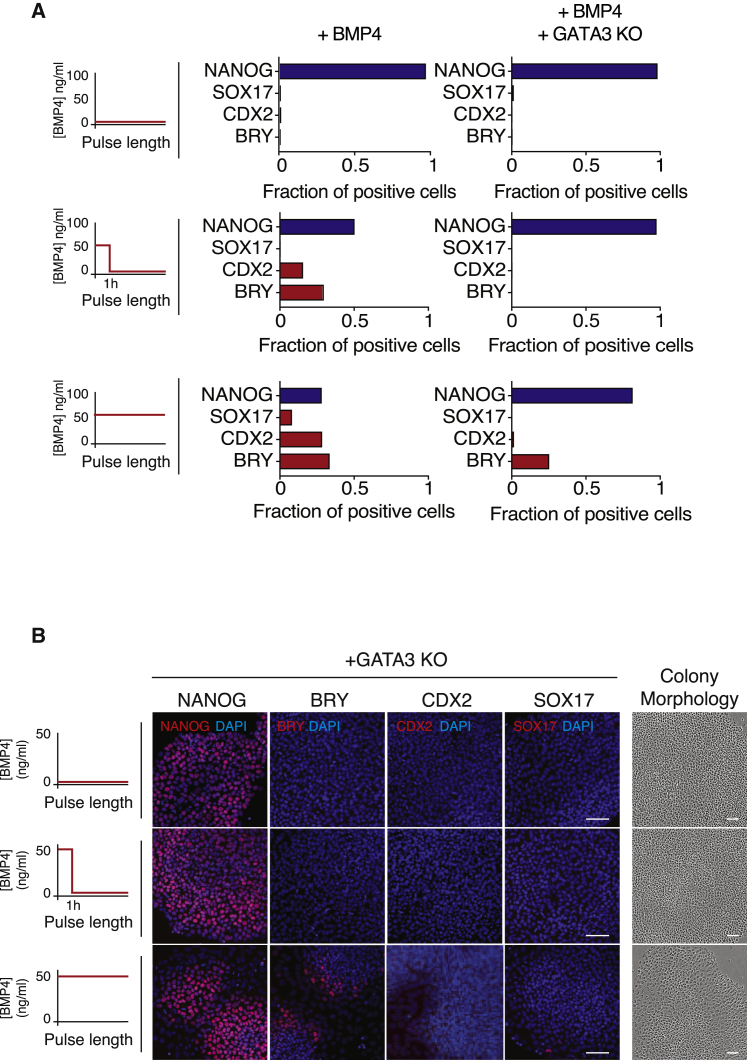
GATA3 Is an Early Commitment Gene, Necessary for Fast Commitment after a Pulse of BMP4 (A) Left: schematic of the duration of BMP4 pulses used to drive hESC differentiation. Right: quantification of the fraction of positive cells showing the expression of pluripotency genes (NANOG, SOX2, and OCT4) and mesoderm-specific genes (BRY, CDX2, and GATA4) at day 2 following different pulses of BMP4 treatment in GATA3^−/−^ (GATA3 KO) cells (red) or control cells (blue). n > 500 cells were analyzed for each experimental condition. (B) Left: schematic of the duration of BMP4 pulses used to drive hESC differentiation. Center: representative images of pluripotency (NANOG) and lineage-specific markers (BRY, CDX2, and SOX17) in response to a pulse of BMP4 (50 ng/mL) for 60 min in GATA3^−/−^ (GATA3 KO) cells. Cells cultured with or without BMP4 for the length of the experiment were used as controls. Images are shown as the merge image between the gene of interest (red channel) and DAPI (blue channel). Scale bar represents 50 μm. Right: representative images of colony morphology for the different experimental conditions. Scale bar represents 50 μm.

To test this, we challenged GATA3 KO hESCs with either a short, 60 min pulse of BMP4 (followed by washing and culturing cells back in mTeSR) or with sustained BMP4 stimulation over 72 h. As seen previously (Figure 1B), a pulse of BMP4 in control hESCs causes the downregulation of pluripotent genes and the upregulation of lineage-specific markers, both hallmarks of cellular differentiation (Figures 6A and 6B). Cells in which GATA3 expression had been compromised failed to differentiate after this short, 60 min pulse of BMP4 (Figures 6A and 6B). In these cells, a short pulse of BMP4 does not lead to NANOG downregulation or upregulation of lineage-specific markers. Instead, these cells resemble pluripotent, control cells (Figures 6A and 6B). Similar results were obtained using GATA3 KD (short hairpin GATA3 [shGATA3]) hESC line (Figures S6A and S6B), showing that perturbing GATA3 expression delays commitment to BMP-induced differentiation.

These observations support the idea that GATA3 is an early commitment gene, whose expression is essential for and mediates early exit from pluripotency and commitment to BMP4-driven differentiation.

### Early Commitment Downstream of BMP Signals Is Likely a Feature of Early Human Development

We sought to understand whether early commitment to differentiation and the function of GATA3 downstream of BMP4 were likely to be a property of fate choice in early human development.

**Figure 7:**
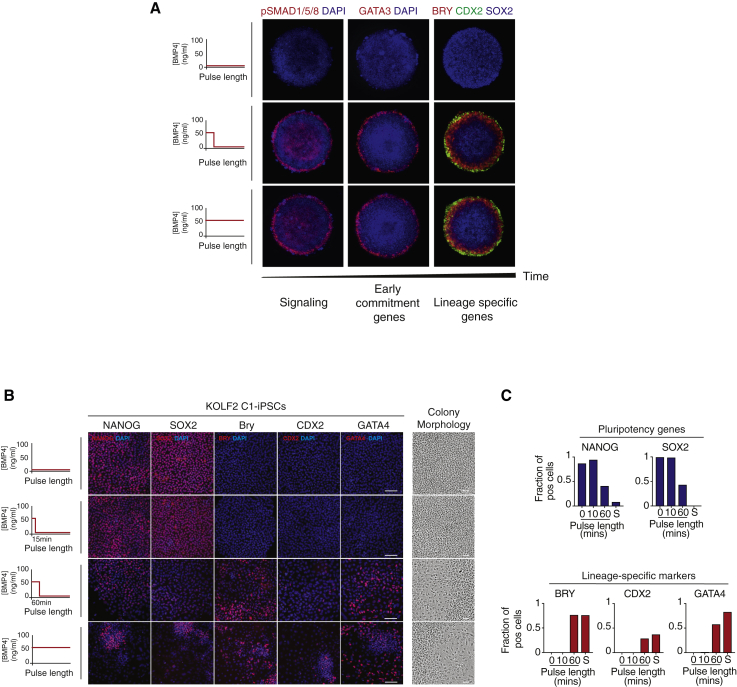
Early Commitment to hESC Differentiation in Gastruloids and IPS Cells (A) Left: schematic of the duration of BMP4 pulses used to drive hESC differentiation. Right: representative images of hESCs growing on micropatterns showing active SMAD (pSMAD 1/5/8), GATA3, and tri-lineage specific markers (BRY, CDX2, SOX2) after 1 and 72 h of BMP4 (50 ng/mL) treatment, respectively. Micropattern diameter 500 μm. (B) Left: schematic of the duration of BMP4 pulses used to drive differentiation in KOLF2-C1 iPS cells. Center: representative images of pluripotency markers (NANOG and SOX2) and lineage-specific markers (BRY, CDX2, and GATA4) in response to 15 min and 60 min pulses of BMP4 stimulation (50 ng/mL). Cells cultured without BMP4 and with sustained (S) BMP4 were used as controls. Images are shown as the merge image between the gene of interest (red channel) and DAPI (blue channel). Scale bar represents 100 μm. Right: representative images of colony morphology for the different experimental conditions. Scale bar represents 50 μm. (C) Quantification of the fraction of positive cells showing the expression of pluripotency (NANOG and SOX2) and mesoderm-specific (BRY, CDX2, and GATA4) genes at day 3 following different pulses of BMP4. n > 500 cells were analyzed for each experimental condition.

To address this, we used an established *in vitro* model of human gastrulation, based on micropatterning cultures of hESCs (Warmflash et al., 2014). We see that a short pulse of BMP4 (followed by washing and culturing cells in mTeSR) is sufficient to activate SMAD1/5/8, to increase GATA3 expression and drive proper tri-lineage patterning (BRY, CDX2, SOX2) of the gastruloid 3 days later, similar to our previous observations of sustained BMP4 stimulation of hESCs (Figure 7A). We further tested whether early commitment could be observed in induced pluripotent stem cells (iPSCs). In agreement with our observations in hESCs, we see that a pulse of BMP4 is sufficient to induce the differentiation of KOLF2-C1 iPS cells (Figures 7B and 7C). This suggests that early commitment is recapitulated in iPS cells and in an established *in vitro* model of early human development.

### Early Pluripotency Exit Is Conserved in BMP4-Induced Fate Choices

We next wondered whether early commitment to differentiation was a conserved feature of BMP4-driven differentiation. To test whether these features are conserved in other fates, hESCs were differentiated with established protocols (see Method Details) into endoderm, mesoderm, trophectoderm, and ectoderm (Figure S7A). We see that a pulse of instructive triggers for distinct progenitor fates (mesoderm, endoderm, and trophectoderm, but not ectoderm) induced a lineage-specific gene signature identical to sustained stimulation (Figures S7B). Notably, all of these protocols contained some level of BMP4, showing that early irreversible commitment may be a conserved property of BMP4-induced fate choices. Comparing early transcriptome signatures for these other fate inductions by RNA-seq analysis showed that early genes, including GATA3, MSX2, GATA2, ID1, and HES1, are shared between endoderm, mesoderm, and trophectoderm fates, and that these have the same early switch-like expression dynamics (Figure S6C) as seen for mTeSR-BMP4-driven differentiation.

## Discussion

These results show that while hallmark events in hESC differentiation happen days after cells first see BMP differentiation cues, commitment to exit pluripotency is surprisingly fast (Figure 1).

Here, we provide a mechanistic model for the early, irreversible commitment to leave the pluripotency state and undergo differentiation triggered by BMP4 signals. We suggest that BMP4 activates early genes, such as GATA3, that respond to and mirror the activation dynamics of upstream signal transduction networks (Figures 2 and 3). The induction of GATA3 above a threshold marks the point of no return to exit pluripotency and undergo differentiation (i.e., after this point, the probability of going back and remaining a pluripotent cell is very low), not only for the subset of cells that expresses it but also for the hESC colony as a whole.

The expression of GATA3 leads to the upregulation of endogenous BMP4 and endows hESCs with the memory of commitment to exit pluripotency (Figure 4). Inducing GATA3 in the absence of differentiation signals drives differentiation, and its absence both dramatically delays differentiation and shifts the point of commitment (Figure 5).

BMP4 is an important instructive cue known to drive mesoderm and cardiac formation (Dale et al., 1992, Winnier et al., 1995, Bernardo et al., 2011), and GATA3 co-localizes with canonical mesoderm markers, including BRY, MIXL1, GSC, and MESP1 (Figures 5F and 5G). The lack of mesoderm formation in BMP4 KO mice highlights its essential role in early gastrulation.

We therefore suggest that despite gastrulation being a dramatic process in the development of an embryo, the initial decision to undergo this process may be established much earlier than anticipated. We see that this initial decision depends on the integration of the strength and duration of BMP4 signals (Figures S2D and S7D), which allows cells to reach thresholds of activities and undergo differentiation. As such, for high BMP4, a short pulse of BMP4 stimulation is sufficient to induce hESC differentiation, whereas a longer pulse is needed if the BMP4 concentration is below threshold concentrations (Figure S7D).

As seen in other all-or-none cellular transitions (Xiong and Ferrell, 2003, Santos et al., 2007, Santos et al., 2012, López-Avilés et al., 2009;), interlinked feedback regulation ensures bistability in the signaling and gene expression networks that transduce BMP4 signals (Figures 2 and 3). This feedback control, coupled with changes in nuclear import and export rates upon BMP4 stimulation (Schmierer and Hill, 2005), is likely to promote the short-term memory of SMAD activation. In addition, the upregulation of transforming growth factor β (TGF-β) inhibitors upon BMP4 stimulation (e.g., BAMBI, SMAD7) (Figures S3A and S3B) suggests that there may be long-term cross-inhibition between differentiation and pluripotency networks. This potential double-negative feedback is a motif that has been shown to underlie points of no return in fate transitions and lock cellular states (Huang et al., 2007). These interlinked feedbacks could ensure memory and irreversibility in the pluripotency to differentiation transition. BMP4-mediated feedback control has indeed been shown to be key in other classical developmental systems (Biehs et al., 1996, Kim et al., 1998).

This study sheds light on how cells decode signals and irreversibly exit pluripotency and commit to differentiation. In this regard, hESCs can be a valuable model system for understanding the molecular mechanisms underlying fate decisions during human early development.

## STAR★Methods

### Key Resources Table

**Table:** 

REAGENT or RESOURCE	SOURCE	IDENTIFIER
**Antibodies**		

GATA3 (rabbit, monoclonal) (IF, ChIP, WB)	Abcam	ab199428; RRID:AB_2819013
SMAD1 (rabbit, monoclonal) (ChIP)	Cell Signaling Technology	6944; RRID:AB_10858882
BRACHYURY (goat, polyclonal) (IF)	R&D Systems	AF2085; RRID:AB_2200235
CDX2 (rabbit, monoclonal) (IF)	Abcam	ab76541; RID:AB_1523334
GATA3 (rabbit, monoclonal) (IF)	Cell Signaling Technology	5852; RRID:AB_10835690
GATA4 (rabbit, monoclonal) (IF)	Cell Signaling Technology	36966; RRID:AB_2799108
NANOG (rabbit, monoclonal) (IF)	Cell Signaling Technology	4903; RRID:AB_10559205
NANOG (mouse, monoclonal) (IF)	Cell Signaling Technology	4893; RRID:AB_10548762
Phospho-SMAD1/5/9 (rabbit, monoclonal) (IF)	Cell Signaling Technology	13820; RRID:AB_2493181
Phospho-SMAD1/5 (rabbit, monoclonal) (IF)	Cell Signaling Technology	9516; RRID:AB_491015
SOX2 (rabbit, monoclonal) (IF)	Cell Signaling Technology	3579; RRID:AB_2195767
SOX17 (rabbit, monoclonal) (IF)	Cell Signaling Technology	81778; RRID:AB_2650582
Alpha-Tubulin (mouse, monoclonal) (WB)	Sigma	B512; RRID:AB_86546

**Chemicals, Peptides, and Recombinant Proteins**		

Recombinant human BMP4	Life Technologies	PHC9534
FGF-Basic (AA 10-155) Recombinant Human Protein	Life Technologies	PHG0026
Human recombinant VEGF	Life Technologies	PHC9394
Activin A	Cambridge Bioscience	GFH6-10
LY294002	Cambridge Bioscience	SM24-10
Poly-L-Lysine-g-Polyethylene Glycol	SuSOS	PLL(20)-g[3.5]- PEG(5)
iMatrix Laminin 511	Takara	T304
Noggin	R&D Systems	6057-NG-025/CF
K02288	Selleckchem	S7359-SEL
ROCK inhibitor Y-27632	Merck	509228
Z(4)-hydroxytamoxifen (4-OHT)	Sigma	H7904
Doxycycline hydrochloride	Sigma Aldrich	D9891
Actinomycin D	Sigma Aldrich	A4262
Cycloheximide	Sigma Aldrich	46401
Puromycin dihydrochloride	GIBCO	12122530

**Critical Commercial Assays**		

STEMdiff Mesoderm Induction Medium	STEMCELL Technologies	05221
Affymetrix QuantiGene ViewRNA ISH Cell Assay Kit	Thermo Fisher Scientific	QVC0001
Human BMPR1A View Type 4 RNA ISH Probe	Thermo Fisher Scientific	VA4-20909
Human GATA3 View Type 4 RNA ISH Probe	Thermo Fisher Scientific	VA4-16345
Genome-CRISP Inducible Cas9 human AAVS1 Safe Harbor knockin Kit	GeneCopoeia	SH016

**Deposited Data**		

RNA-Seq: H1 hESC (0, 20, 40, 60 mins BMP4)	This paper	GEO: GSE127936
RNA-Seq: H1 hESC (0, 20, 40, 60 mins Lineage-specific differentiation; Troph/Meso/Endo)	This paper	GEO: GSE127935
ChIP-Seq: SMAD1 in H1 hESC (0, 60 mins BMP4)	This paper	GEO: GSE135254
ChIP-Seq: GATA3 in H1 hESC (6 hr BMP4)	This paper	GEO: GSE135255
scRNA-Seq: H1 hESC (48 hr BMP4)	This paper	GEO: GSE135253

**Experimental Models: Cell Lines**		

H1 hESC (WA01)	WiCell	RRID:CVCL_9771
H9 hESC (WA09)	WiCell	RRID:CVCL_9773
KOLF2 C1 hiPSC	HipSci	RRID:CVCL_9S58
HEK293T	ATCC	RRID:CVCL_0063
H1 hESC Tet-inducible SpCas9	This paper	CAS001
H1 hESC GATA3 Knockout	This paper	KO-GATA3-06

**Oligonucleotides**		

*All primers for qPCR, ChIP-qPCR, and Screening are listed in*Table S1	This paper	N/A

**Recombinant DNA**		

Plasmid: pCSII EF1α GFP-T2A-ERT-hGATA3	This paper	N/A
Plasmid: pRRL GEP hGATA3 shRNA #1	This paper; Fellmann et al., 2013	N/A
Plasmid: pRRL GEP hGATA3 shRNA #2	This paper; Fellmann et al., 2013	N/A
Plasmid: pRRL GEP hGATA3 shRNA #3	This paper; Fellmann et al., 2013	N/A

**Software and Algorithms**		

MATLAB	MathWorks	https://uk.mathworks.com
Integrative Genomics Viewer	Robinson et al., 2011, Thorvaldsdóttir et al., 2013	https://software.broadinstitute.org/software/igv/
Fiji	Schindelin et al., 2012	https://imagej.net/Fiji
TopHat2	Kim et al., 2013	http://ccb.jhu.edu/software/tophat
Cell-Ranger 3.0.2	10x Genomics	https://www.10xgenomics.com/
Seurat 3	Stuart et al., 2019	https://rdrr.io/cran/Seurat/
FASTQC	Wingett and Andrews, 2018	https://www.bioinformatics.babraham.ac.uk/projects/fastqc/
Burrows-Wheeler Aligner (BWA)	Li and Durbin, 2009	http://bio-bwa.sourceforge.net/
MACS/MACS2	Zhang et al., 2008	https://taoliu.github.io/MACS/
ChIPQC	Carroll et al., 2014	https://bioconductor.org/packages/release/bioc/html/ChIPQC.html
DeSeq2	Love et al., 2014	https://bioconductor.org/packages/release/bioc/html/DESeq2.html
DAVID	(Huang et al., 2009a, Huang et al., 2009b)	https://david.ncifcrf.gov/
Cytoscape	Cytoscape Consortium	https://cytoscape.org/

### Lead Contact and Materials Availability

Enquiries on reagents and resources should be directed to, and will be fulfilled by the lead contact Dr Silvia Santos (silvia.santos@crick.ac.uk). Plasmids generated in this study will be made freely available upon request. Modified human embryonic stem cell lines generated in this study will be made available on request but will require a completed Materials Transfer Agreement from WiCell.

### Experimental Model and Subject Details

All experiments were performed using either wild-type human embryonic stem (hES) cell lines H1 (XY; WA01, WiCell; RRID:CVCL_9771) and H9 (XX; WA09, WiCell; RRID:CVCL_9773), originally derived by the Thomson lab (Thomson et al., 1998), or induced pluripotent stem cells (iPS) KOLF2 C1 (XY; from Human induced pluripotent stem cell initiative, HipSci, www.hipsci.org; RRID:CVCL_9S58). Genome-edited monoclonal lines were generated during this study from cell line H1. H1, H9, and KOLF2 cell lines were routinely cultured in serum-free, feeder-free conditions, on growth factor reduced (GFR) Matrigel-coated plates. Cells were fed daily using chemically defined medium (mTeSR1, STEMCELL Technologies), were incubated at 37°C with 5% CO2 and passaged every 3-4 days. Gentle dissociation buffer (STEMCELL Technologies) was used for passaging. Cells were routinely screened for mycoplasma. Cell lines were authenticated by karyotyping.

Lentivirus was generated in HEK293T cells (XX; ATCC Cat# CRL-3216; RRID:CVCL_0063), which were maintained in DMEM (GIBCO) with 10% Foetal Bovine Serum, at 37°C with 5% CO2 and passaged once every 4-5 days by dissociation using TrypLE Express Enzyme (Thermo Fisher).

### Method Details

#### Differentiation of hES cells in chemically defined conditions

For typical differentiation experiments (marked by expression of Brachyury, CDX2 and GATA4), cells were treated with recombinant human BMP4 (GIBCO) at 50 ng/ml in mTeSR1, unless otherwise specified. For alternative mesoderm protocol I, conditions from Evseenko et al. 2010) were utilized: cells were stimulated with 10 ng/ml BMP4 (GIBCO), 10 ng/ml bFGF (Life Technologies), 10 ng/ml VEGF (ThermoFisher Scientific) and 10 ng/ml Activin (Cambridge Bioscience) in TeSR-E5 media (STEMCELL Technologies). For alternative mesoderm protocol II, STEMdiff Mesoderm Induction Medium was used, a commercially available kit for mesoderm induction (STEMCELL Technologies). To differentiate cells toward the endoderm lineage, cells were cultured in TeSR-E5 media (STEMCELL technologies) in the presence of 10 ng/ml BMP4 (GIBCO), 20 ng/ml bFGF (Life Technologies), 100 ng/ml Activin A (Cambridge Bioscience;) and 10 μM LY294002 (Cambridge Bioscience) as described in Bernardo et al. (2011). For differentiation toward the ectoderm lineage, cells were cultured in TeSR-E5 media supplemented with 200 ng/ml Noggin (R&D systems), 10 μM SB-431542 (Tocris) and 12 ng/ml bFGF (Life Technologies), as described in Chambers et al. (2009). For trophoblast differentiation, cells were stimulated with 50 ng/ml BMP4 in the absence of FGF using TeSR-E5 media. For Gata3 induction (iGATA3) experiments, cells were stimulated with 1 μM 4OHT in the presence of TeSR-E5 media.

#### Making and culturing hES cells in 2D confinement using micropatterns

Circular 1 mm micropatterned cell culture surfaces ¬were produced as described in Tewary et al. (2019). In brief, glass coverslips were coated with Poly-L-Lysine-grafted-Polyethylene Glycol (PLL-g-PEG). PEGylated coverslips were then treated with Deep UV (DUV) through Quartz photo-masks for 8 minutes. Following DUV treatment, carboxyl-rich regions were biofunctionalized by extracellular matrix proteins (ECM). Coverslips were then washed and transferred to standard tissue culture plates. Prior to seeding cells, coverslips were coated with recombinant laminin (iMatrix Laminin 511, Takara) at 0.5 mg/ml in PBS for 2 hours at 37°C. hES cells were then seeded as single cells onto micro-patterned coverslips as described previously by Warmflash et al. (2014) and maintained in mTeSR1 medium. Following overnight incubation, cells were stimulated with 50 ng/ml BMP4 in mTeSR1 and fixed either at 1 hour for pSMAD1/5 staining, 8 hours for GATA3 staining or 48 hours for SOX2 and Brachyury staining.

#### Small Molecule Inhibitors

The inhibitors used in this study were: Noggin (at 250 ng/ml, R&D systems), K02288 (at 1 μM, Selleckchem), ROCK inhibitor Y-27632 (at 10 μM, Calbiochem), PI3K inhibitor (LY294002 at 10 μM, Cambridge Bioscience), TGFβ receptor inhibitor (SB-431542 at 10 μM, Tocris), Actinomycin (added for 1 hour at 5 μg/ml, SLS) and Cycloheximide (added for 4 hours at 50 μg/ml, SLS) unless otherwise specified.

#### Generating inducible GATA3 hES cells

To control GATA3 expression in an inducible fashion, a mutant estrogen receptor was N-terminally fused to GATA3 (ERT-GATA3), which enables translocation of exogenous GATA3 protein by addition of Z(4)-Hydroxytamoxifen (4-OHT, Sigma; #H7904). ERT-GATA3 was cloned into the lentiviral vector pCSII-EF1α-MCS-2 by restriction digestion and ligation reactions. Stable lines expressing ERT-GATA3 were produced by lentiviral infection.

#### Generating GATA3 knockdown (KD) hES cell line using shRNA

For knockdown experiments, predicted shRNA sequences against human GATA3 5′-TGCTGTTGACAGTGAGCGACGGGCTCTATCACAAAATGAATAGTGAAGCCACAGATGTATTCATTTTGTGATAGAGCCCGCTGCCTACTGCCTCGGA-3′ and 5′-TGCTGTTGACAGTGAGCGCCCGAACTGTTGTATAAATTTATAGTGAAGCCACAGATGTATAAATTTATACAACAGTTCGGTTGCCTACTGCCTCGGA-3′ were subcloned into the lentiviral SGEP vector (containing GFP) using XhoI/EcoRI enzymes and Gibson cloning strategy. Fluorescence-coupled shGATA3 was expressed downstream of an optimized Tet-responsive element promoter (TRE3G) allowing inducible expression in response to the addition of doxycycline hydrochloride. Stable lines expressing shGATA3 were produced by lentiviral infection. Predicted sequences and pRRL SGEP vectors are described in Fellmann et al. (2013).

#### Lentiviral Transduction

To generate lentivirus, HEK293T cells were seeded at ∼50% density. The following day, growth media was removed and replaced with mTeSR1. Cells were then transfected with the lentiviral packaging plasmids psPAX2 (Addgene #12260), pMD2.G (VSV-G expressing; Addgene #12259), and the construct of interest in a 4:1:5 ratio (μg DNA) via polyethylenimine (PEI; 1:3 μg DNA: μg PEI). Media was harvested 2-3 days after transfection. hESC were infected with virus-containing media and 10 μg/ml polybrene for 24 hours.

#### Generating GATA3−/− knock-out (KO) hES cell line using CRISPR-Cas9

H1 hES cells were used to generate a monoclonal line with an integrated doxycycline-inducible SpCas9 expression cassette; briefly, cells were transfected with plasmids from the Genome-CRISP Inducible Cas9 human AAVS1 Safe Harbor Knockin Kit (GeneCopoeia) using Fugene HD (Promega) and selected with Puromycin (500 ng/ml), then deposited as single cells into a Matrigel-coated 384-well tray using FACS and grown in mTeSR1 with CloneR supplement as per manufacturer’s recommendations (STEMCELL Technologies). One of the resulting monoclonal lines (CAS001) was transfected with tracrRNA and crRNA against human GATA3 (Dharmacon; GATA3 crRNA01 and GATA3 crRNA03) using Lipofectamine RNAiMAX (Invitrogen) and treated with doxycycline (1 μg/ml) to induce SpCas9 expression. Monoclonal lines were grown as described above, and KO clones were identified by immunofluorescent staining, PCR, and Sanger sequencing. Both the parental SpCas9 line (CAS001) and the GATA3 KO line used in this paper (KO-G3-06) were confirmed to be karyotypically normal.

#### Quantitative real-time PCR (qPCR)

RNA was extracted using TRI reagent (Life Technologies) and MaXtract High Density tubes (QIAGEN) according to the manufacturer’s instructions. For cDNA preparation and DNA elimination, the QuantiTect Reverse Transcription Kit (QIAGEN) was used according to the manufacturer’s instructions. cDNA was prepared from 1.25 μg RNA. RNA purity and quantity was assessed by Nanodrop (A260/A280 1.8-2 was considered suitable for further analysis). qRT-PCR was performed on a Bio-Rad CFX96 real-time detection system using iTaq SYBR Green qPCR master mix (Bio-Rad). PCR conditions consisted of 1 cycle of 95°C for 3 min and 40 cycles of 95°C for 5 s and 60°C for 30 s. The housekeeping gene Glucuronidase beta (GUSB) was used as a normalization control.

#### Western Blotting

Cells were lysed with RIPA Buffer. Proteins were separated on a 10% Criterion TGX Gel (BioRad) and transferred to PVDF membrane before blocking with 5% Skim Milk in TBST (Tris-buffered Saline, 0.1% Tween-20). Primary antibodies used were against GATA3 (Abcam, ab199428) and alpha-Tubulin (Sigma, B512), secondary antibodies used were goat anti-rabbit HRP (Cayman Chemical) and IRDye 800CW goat anti-mouse (LI-COR, 926-32210). Chemiluminescence was imaged using an Amersham Imager 600 (GE), and fluorescence was imaged using an Odyssey CLx (LI-COR).

#### Immunofluorescence (IF)

Cells were washed briefly with cold PBS to halt reactions and fixed using 4% formaldehyde in PBS for 10 minutes at room temperature. Cells were washed with PBS and permeabilized using permeabilization buffer (0.3% Triton X-100 in PBS) for 10 minutes at room temperature. Cells were then washed with PBS and incubated with blocking buffer (10% fetal bovine serum and 3% bovine serum albumin in PBS) for 1 hour at room temperature prior to incubation with primary antibodies at 4°C overnight in primary antibody dilution buffer (1% bovine serum albumin and 0.3% Tween in PBS). Following overnight incubation with primary antibodies, cells were washed (0.3% Tween in PBS), and incubated with secondary antibodies in blocking buffer for 1 hour at room temperature. Cells were washed again and nuclei were stained using DAPI. Primary antibodies used for IF were phospho-SMAD1/5 (Cell Signaling Technology; 9516), phospho-SMAD1/5/9 (Cell Signaling Technology; 13820), BRY (R&D Systems; AF2085), CDX2 (Abcam, ab76541), GATA3 (Abcam, ab199428 OR Cell Signaling Technology; 5852), GATA4 (Cell Signaling Technology; 36966), NANOG (Cell Signaling Technology; 4903 OR 4893), SOX2 (Cell Signaling Technology; 3579), and SOX17 (Cell Signaling Technology; 81778). Secondary antibodies were from the Alexa Fluor series (Life Technologies).

For dose response experiments, cells were fixed at steady state. In the case of SMAD activation steady state was considered as 60 minutes following BMP4 stimulation and for GATA3 expression as 120 minutes. For dose response experiments testing different pulses of BMP4 cells were fixed and stained at 72h.

For experiments to test phospho-SMAD1/5/8 and GATA3 irreversibility, cells were stimulated with BMP4 (50 ng/ml) followed by addition of Noggin (100 ng/ml) to inhibit BMP4 signaling prior to, and following the peak in SMAD activation. For inhibition after the peak, cells were stimulated with BMP4 (50 ng/ml) for 30 minutes, then washed with PBS and incubated with Noggin (100 ng/ml). For inhibition prior to the peak, cells were stimulated with BMP4 (50 ng/ml) for 15 minutes to initiate activation of the SMAD signaling pathway, after which Noggin was added. Cells were fixed and stained after 60 min stimulation for phospho-SMAD1/5/8 and after 12 hours for GATA3 to reach steady-state conditions.

#### Single molecule RNA-FISH

RNA FISH and combined RNA FISH and immunofluorescence was performed using the Affymetrix QuantiGene ViewRNA ISH Cell Assay kit (Thermofisher) according to the manufacturer’s instructions. Probes for GATA3 and BMPR1A were acquired from Affymetrix (Thermofisher).

#### Microscopy

For live cell microscopy, hES cells were typically seeded into 96-well glass-bottom imaging plates (Greiner Bio-One Ltd) and allowed to settle and attach prior to commencing the experiment. hES colonies were allowed to reach a critical size before stimulation.

Imaging was performed on either a ScanR - a fully motorized and automated inverted epifluorescence microscope system IX83 (Olympus) - combined with CellVivo (Olympus), or an IncuCyte Zoom® (Essen BioScience). Both were equipped with temperature, humidity and CO2 levels control to maintain sample integrity and perfect focus. ScanR images were typically acquired with a 20x plan (UCPLFLN) fluorescence objective (NA 0.7) and a sCMOS (Orca Flash 4.0, Hamamatsu) camera. LED-based illumination (SpectraX LED, Lumenco) was used for excitation. Excitation (ex) and emission (em) filters were as follows: DAPI ex: 391/20nm, em: 440/521/607/700nm; GFP/Alexa 488 ex: 474/27nm, em: 440/521/607/700nm and mCherry ex: 554/23nm, em: 440/521/607/700nm. IncuCyte Zoom images were acquired with either 4x, 10x or 20x plan fluorescence objectives and a CCD camera. Fluorescence excitation (ex) and emission (em) filters were as follows: Green channel ex: 440-480nm, em: 504-544nm; Red channel ex: 565-605nm em: 625-705nm. False color and merged-channel images were generated using Fiji (Schindelin et al., 2012).

#### RNA-Seq

H1 hES cells were treated with BMP4 (50 ng/ml, GIBCO) in mTeSR1 or with different fate cocktails for 20, 40 and 60 minutes before cells were washed with PBS and total RNA was extracted using RNeasy mini kit (QIAGEN) as per the manufacturer’s instructions. Three biological replicates were made for each experimental condition. The quality and quantity of RNA extracted was tested using a 2100 Bioanalyzer instrument (Agilent Genomics).

For the initial study (H1 hESC treated with BMP4 for 0, 20, 40, 60 mins), sequencing libraries were prepared using the Truseq stranded mRNA library prep kit (Illumina) starting from 500 ng of total RNA and following manufacturer’s protocol. Sequencing was performed using a HiSeq2500 system (Illumina) using paired end 100 bp reads. The Ensembl hg19 human genome build and respective gene models were retrieved from IGenomes. RNA-seq raw reads were aligned to this build using Tophat version 2.0.11 (Kim et al., 2013) and counted using featureCounts in the Rsubread version 1.16 Bioconductor package.

For the follow-up study, in which H1 hESCs were treated with cocktails to differentiate toward mesoderm, endoderm, or trophectoderm (0, 20, 40, 60 mins), libraries were prepared using a KAPA mRNA HyperPrep Kit following manufacturer’s protocol. Sequencing was performed using a HiSeq4000 system (Illumina) using 75 bp single end reads. RNA-Seq libraries were sequenced to a mean depth of 36.6 million reads (sd 4.7). Raw reads were quality and adaptor trimmed using cutadapt-1.9.1 (Martin, 2011) prior to alignment. Reads were aligned and quantified using RSEM-1.3.0/STAR-2.5.2 (Dobin et al., 2013; Li and Dewey, 2011) against the human genome GRCh38, annotation release 86, from Ensembl. Alignment rates were all > 98% with a mean number of 21370 genes detected. The mean duplication rate was 67% (Picard MarkDuplicates).

#### Single cell RNA-Seq

Single cell suspensions of BMP4 treated (48h) H1 hES cells (500-750 cells/μl in PBS+BSA with > 95% viability) were prepared by treating cells with gentle dissociation buffer and passing cells through a cell strainer. The quality and concentration of each single cell suspension was measured using Trypan blue and the Eve automatic cell counter. Each sample was diluted to a concentration of ∼1000 cells/μl and approximately 10000 cells were loaded for each sample into a separate channel of a Chromium Chip B for use in the 10X Chromium Controller. The cells were partitioned into nanoliter scale Gel Beads in emulsions (GEMs) and lysed using the Chromium Single cell 3′ v3 GEM, Library and Gel Bead Kit (cat: 1000075). The RNA was reversed transcribed and amplified using 11 cycles of PCR. Libraries were prepared from the cDNA using a further 12 cycles of amplification and sequenced on the HiSeq4000 system (Illumina). The library was sequenced to a depth of 661,709,731 (WT) and 639,936,775 (KO) reads. We used Cellranger version 3.0.2 to align the reads to human transcriptome GRCh38-3.0.0 (software and data available from 10x genomics). From these alignments, we were able to identify 3,744 high quality cells with a mean reads per cell value of 111,517 and a median genes per cell count of 6,551 (WT) and 6,487 (KO). The total number of reads and number of detectable genes are comparable between these two samples so the differences we are seeing are unlikely to be due to differences in the sampling of the libraries.

#### ChIP-qPCR

For ChIP-qPCR, H1 hES cells were grown in mTeSR with or without BMP4 (50 ng/ml, GIBCO) for 1 hour or 6 hours as indicated, before being dissociated with TrypLE Express Enzyme (GIBCO) and fixed in mTeSR1 media with 1% methanol-free formaldehyde (Pierce) for 10 minutes at room temperature. Fixation was quenched with 125 mM Glycine and cells were washed with cold PBS before being resuspended in 300 μL of High Salt Sonication Buffer (800 mM NaCl, 25 mM Tris, 5 mM EDTA, 1% Triton X-100, 0.1% SDS, 0.5% Sodium deoxycholate, Protease Inhibitors). Chromatin from approximately 3E+06 cells per ChIP was sheared using a Bioruptor Plus (Diagenode) to lengths of 100-400 bp, and then diluted 1:4 with Chromatin Dilution Buffer (25 mM Tris, 5 mM EDTA, 1% Triton X-100, 0.1% SDS, Protease Inhibitors). Primary antibody was incubated with 20 μL of Protein G Dynabeads (Invitrogen) at room temperature for 3 hours. Antibody with bead slurry was added to sheared chromatin and incubated with rotation at 4°C overnight. Beads were then washed at 4°C for 5 mins with 1 mL of (1) Wash Buffer A (50mM HEPES, 140mM NaCl, 1mM EDTA, 1% Triton X-100, 0.1% Sodium deoxycholate, 0.1% SDS, adjusted to pH 7.9), (2) Wash Buffer B (50 mM HEPES, 500 mM NaCl, 1 mM EDTA, 1% Triton X-100, 0.1% Sodium deoxycholate, 0.1% SDS, adjusted to pH 7.9), (3) Wash Buffer C (20 mM Tris, 1 mM EDTA, 250 mM LiCl, 0.5% NP-40, 0.5% Sodium deoxycholate, adjusted to pH 8.0), (4) TE Buffer (10 mM Tris, 1 mM EDTA), (5) TE Buffer. Complexes were eluted twice and pooled by adding 100 uL Elution Buffer (10 mM Tris, 1 mM EDTA, 1% SDS) and incubating at 65°C for 5 mins, followed by incubation with rotation at room temperature for 15 mins. Pooled eluates were increased to 160mM NaCl and incubated overnight with RNase A (20 μg/ml) at 65°C to reverse crosslinks. Samples were increased to 5mM EDTA and incubated with Proteinase K (200 μg/ml) at 45°C for 2 hours to digest proteins. DNA was purified using ChIP DNA Clean and Concentrator Zymo-Spin Kit (Zymo).

Antibodies used were SMAD1 (Cell Signaling Technology; CST6944) or GATA3 (Abcam, ab199428). qPCRs for ChIP-qPCR experiments were performed with iTaq Universal SYBR Green Supermix (BioRad) on three biological replicates.

#### ChIP-Seq

ChIPs were performed as for ChIP-qPCR on approximately 3E+06 cells grown in mTeSR1 with or without BMP4 (50 ng/ml) for 1 hour (SMAD1 ChIPs) or 6 hours (GATA3 ChIPs). Primary antibodies used were SMAD1 (Cell Signaling Technology, CST6944) and GATA3 (Abcam, ab199428).

For the SMAD1 ChIPs, 2 ng of purified DNA was used to prepare libraries using the NEBNext® Ultra II DNA Library Prep Kit for Illumina following the manufacturer recommendations (15 cycles of PCR). Library quality and quantity were assessed on a Bioanalyser and Qubit, respectively. Libraries were sequenced on an Illumina Hiseq2500 (v4 chemistry) on a Single Read 50 bp run (2 lanes). Sequence quality of the raw reads was evaluated using FASTQC tool (Wingett and Andrews, 2018). Raw reads were aligned to human genome (hg19 assembly) using BWA version 0.7.5a and default settings. The SMAD ChIP and input samples had a mean aligned read count of 83 million reads. Replicate samples for ChIPs and inputs were merged prior to peak calling to improve signal. Peak calling was carried out using MACS version 1.4.2 with default settings. 435 and 413 peaks were detected in untreated and 1h BMP4 samples respectively, with a FRIP value of > 1%. These values include peaks that were determined to be non-specific enrichment of tRNA genomic regions. Normalized genome-wide coverage files were obtained by scaling each sample using total number of mapped reads. Overall quality of the ChIP-Seq experiment was evaluated using ChIPQC Bioconductor package (Carroll et al., 2014).

For GATA3 ChIPs, libraries were prepared from purified DNA using KAPA Hyper Prep Kit (KAPA Biosystems) and sequenced on an Illumina Hiseq4000. The GATA3 ChIP and input samples had a mean aligned read count of 26.5 million reads. 75bp single-end reads were trimmed for Illumina adapters using cutadapt. Reads were aligned to human genome (hg38 assembly) using bowtie (very sensitive). Duplicate reads were identified and removed using Picard. Reads mapping to multiple locations and Encode blacklisted regions were also excluded. Enriched regions were identified by comparing ChIP samples to their corresponding input control using MACS2 (-q 0.05, -B,–SPMR) (Zhang et al., 2008) for two biological replicates. 17165 and 13577 peaks were detected in each replicate with a FRIP value of > 5%. Only peaks present in both replicates were used for further analysis. Bigwig tracks were created from scaled bedGraph files generated by MACS2.

Figures for all ChIP-Seq experiments were generated using Integrative Genomes Viewer (Robinson et al., 2011, Thorvaldsdóttir et al., 2013).

#### ODE model

We uncovered three interlinked positive feedback loops induced by BMP4 stimulation of hES cells: a) SMAD- > BMPR- > SMAD; b) GATA3- > BMP4- > GATA3 and c) GATA3 positive auto-regulation. In order to understand whether these could account for the irreversibility in SMAD activation and GATA3 expression, three ordinary differential equations models were used (Figure 1).

#### Figure 1. Schematic showing network topologies used for ODE model

Simulations of the three models allowed us to address whether interlinked feedback regulation could be the molecular mechanism for the early, irreversible commitment to differentiation in hES cells. To methodically characterize the function of the three feedback loops, we first considered the classical linear form of SMAD signaling network (Model 1), without any feedback regulation. We captured the SMAD and GATA3 dynamics with a state of ODEs having biologically significant parameter values (as shown in Supplemental Information) and numerically solved these equations at steady-state for different concentrations of BMP4. We next added one positive feedback loop (SMAD- > BMPR- > SMAD) (Model 2) to find steady state solutions of the simulated ODEs using the parameters described in Table 6. Finally, we introduced all three experimentally measured interlinked positive feedback loops (Model 3). ODEs shown in Table 7 were solved numerically showing multiple solutions at steady-state for a single BMP4 signal. We applied bifurcation analysis on Model 3 equations and found an irreversible bistable switch of active SMAD and GATA3 expression to BMP4 concentration. Each bifurcation diagram contains two stable branches (solid line) and one unstable branch (dotted line). At low concentration of BMP4 (< 10 ng/ml), cell can have either a high or a low population of active SMAD and GATA3. But when BMP4 concentration crosses this threshold (> 10 ng/ml), all cells move to the high stable expression branch. Once a cell attains the high stable state, it never transitions into the low stable branch. These simulations corroborate the experimental observations.

##### Parameters used for model 1

**Table:** 

	**Reaction**	**Parameter value**	**Description**
1.	$\text{BMP}4\;+\text{ BMP}4{\text{R}}_{\text{off}}\to \text{BMP}4\;+\text{ BMP}4\text{R}$	0.04conc^-1^ s^-1^	BMP4 induced BMP4R activation
2.	$\text{BMP}4\text{R}\to \text{BMP}4{\text{R}}_{\text{off}}\text{ff}$	6.0 s^-1^	Deactivation of BMP4R
3.	$\text{BMP}4\text{R }+\text{ SMAD}1_{\text{off}}\to \text{BMP}4\text{R }+\text{ SMAD}1$	0.005conc^-1^ s^-1^	Active BMP4R induced SMAD1 activation
4.	$\text{BMP}4{\text{R}}_{\text{off}}+\text{ SMAD}1_{\text{off}}\to \text{BMP}4{\text{R}}_{\text{off}}+\text{ SMAD}1$	0.0005conc^-1^ s^-1^	Inactive BMP4R induced SMAD1 activation
5.	$\text{SMAD}1\to \text{SMAD}1_{\text{off}}$	0.07 s^-1^	Deactivation of SMAD1
6.	$\overset{\frac{\text{SMAD}1}{\text{SMAD}1+kb3}}{\to }\text{mGATA}3$	0.2conc s^-1^	SMAD1 induced synthesis of GATA3 mRNA
		kb3=25conc	
7.	mGATA3→∅	0.04 s^-1^	Degradation of GATA3 mRNA
8.	mGATA3→GATA3 + mGATA3	0.1 s^-1^	Translation of GATA3
9.	GATA3→∅	0.002 s^-1^	Degradation of GATA3

##### Other Parameters

$$\text{SMAD}1_{\text{off}}+\text{ SMAD1 }=\text{ SMAD1 Total }=\;200\text{conc}$$

$$\text{BMP}4{\text{R}}_{\text{off}}+\text{ BMP4R }=\text{ BMP4R Total }=\;350\text{conc}$$

##### Parameters used for model 2

**Table:** 

	**Reaction**	**Parameter value**	**Description**
1.	$\text{BMP}4\;+\text{ BMP}4{\text{R}}_{\text{off}}\to \text{BMP}4\;+\text{ BMP}4\text{R}$	0.04conc^-1^ s^-1^	BMP4 induced BMP4R activation
2.	$\text{BMP}4\text{R}\to \text{BMP}4{\text{R}}_{\text{off}}$	6.0 s^-1^	Deactivation of BMP4R
3.	$\text{BMP}4\text{R }+\text{ SMAD}1_{\text{off}}\to \text{BMP}4\text{R }+\text{ SMAD}1$	0.005conc^-1^ s^-1^	Active BMP4R induced SMAD1 activation
4.	$\text{BMP}4{\text{R}}_{\text{off}}+\text{ SMAD}1_{\text{off}}\to \text{BMP}4{\text{R}}_{\text{off}}+\text{ SMAD}1$	0.0005conc^-1^ s^-1^	Inactive BMP4R induced SMAD1 activation
5.	$\text{SMAD}1\to \text{SMAD}1_{\text{off}}$	0.0 s^-1^	Deactivation of SMAD1
6.	→mBMP4R	0.0001conc s^-1^	Basal synthesis of BMP4R mRNA
7.	$\overset{\frac{\text{SMAD}1}{\text{SMAD}1+kb1}}{\to }\text{mBMP}4\text{R}$	0.2conc s^-1^$kb1=10^{3}$conc	SMAD1 induced synthesis of BMP4R mRNA
8.	mBMP4R→∅	0.02 s^-1^	Degradation of BMP4R mRNA
9.	$\text{mBMP}4\text{R}\to \text{BMP}4{\text{R}}_{\text{off}}+\text{ mBMP}4\text{R}$	0.1 s^-1^	Translation of BMP4R
10.	BMP4R_off→∅	0.008 s^-1^	Degradation of BMP4R
11.	$\overset{\frac{\text{SMAD}1}{\text{SMAD}1+kb3}}{\to }\text{mGATA}3$	0.2conc s^-1^kb3=25conc	SMAD1 induced synthesis of GATA3 mRNA
12.	mGATA3→∅	0.04 s^-1^	Degradation of GATA3 mRNA
13.	mGATA3→GATA3 + mGATA3	0.1 s^-1^	Translation of GATA3
14.	GATA3→∅	0.002 s^-1^	Degradation of GATA3

##### Other Parameters

$$\text{SMAD}1_{\text{off}}+\text{ SMAD1 }=\text{ SMAD1 Total }=\;200\text{μm}$$

##### Parameters used for model 3

**Table:** 

	**Reaction**	**Parameter value**	**Description**
1.	$\text{BMP}4\;+\text{ BMP}4{\text{R}}_{\text{off}}\to \text{BMP}4\;+\text{ BMP}4\text{R}$	0.04conc^-1^ s^-1^	BMP4 induced BMP4R activation
2.	$\text{BMP}4\text{R}\to \text{BMP}4{\text{R}}_{\text{off}}$	6.0 s^-1^	Deactivation of BMP4R
3.	$\text{BMP}4\text{R }+\text{ SMAD}1_{\text{off}}\to \text{BMP}4\text{R }+\text{ SMAD}1$	0.005conc^-1^ s^-1^	Active BMP4R induced SMAD1 activation
4.	$\text{BMP}4{\text{R}}_{\text{off}}+\text{ SMAD}1_{\text{off}}\to \text{BMP}4{\text{R}}_{\text{off}}+\text{ SMAD}1$	0.0005conc^-1^ s^-1^	Inactive BMP4R induced SMAD1 activation
5.	$\text{SMAD}1\to \text{SMAD}1_{\text{off}}$	0.07 s^-1^	Deactivation of SMAD1
6.	→mBMP4R	0.0001conc s^-1^	Basal synthesis of BMP4R mRNA
7.	$\overset{\frac{\text{SMAD}1}{\text{SMAD}1+kb1}}{\to }\text{mBMP}4\text{R}$	0.2conc s^-1^	SMAD1 induced synthesis of BMP4R mRNA
		$kb1=10^{3}$μm	
8.	$\overset{\frac{\text{GATA}3}{\text{GATA}3+kb2}}{\to }\text{mBMP}4\text{R}$	0.1conc s^-1^	GATA3 induced synthesis of BMP4R mRNA
		$kb2=10^{3}$μm	
9.	mBMP4R→∅	0.02 s^-1^	Degradation of BMP4R mRNA
10.	$\text{mBMP}4\text{R}\to \text{BMP}4{\text{R}}_{\text{off}}+\text{ mBMP}4\text{R}$	0.1 s^-1^	Translation of BMP4R
11.	BMP4R_off→∅	0.008 s^-1^	Degradation of BMP4R
12.	$\overset{\frac{\text{SMAD}1}{\text{SMAD}1+kb3}}{\to }\text{mGATA}3$	0.2conc s^-1^	SMAD1 induced synthesis of GATA3 mRNA
		kb3=25conc	
13.	$\overset{\frac{\text{GATA}3^{2}}{\text{GATA}3^{2}+kb4^{2}}}{\to }\text{mGATA}3$	1.7conc s^-1^	GATA3 induced synthesis of GATA3 mRNA
		kb4=1100conc	
14.	mGATA3→∅	0.04 s^-1^	Degradation of GATA3 mRNA
15.	mGATA3→GATA3 + mGATA3	0.1 s^-1^	Translation of GATA3
16.	GATA3→∅	0.002 s^-1^	Degradation of GATA3

##### Other Parameters

$$\text{SMAD}1_{\text{off}}+\text{ SMAD1 }=\text{ SMAD1 Total }=\;200\text{conc}$$

### QUANTIFICATION AND STATISTICAL ANALYSIS

#### Image Analysis

All cell image data was analyzed using custom-made scripts written in MATLAB. For fixed cell microscopy, DAPI staining was used to mark nuclei in order to create masks for analysis. Mean intensities were used to measure the expression/activity of the protein being tested within masks.

#### RNA-Seq Analysis

For the initial study (H1 hESC treated with BMP4 for 0, 20, 40, 60 mins), identification of genes changing across time and between stages was performed using the DESeq2 version 1.2.10 Bioconductor library (Love et al., 2014). Differential genes were selected by fitting an LRT model across the time-points for each lineage. Genes differential at any one time point were selected using a 0.05 false-discovery rate (FDR) threshold and an absolute log2 fold change > 2 at any one time-point when compared to time zero. Genes changing across time with an adjusted p value (Benjamini-Hochberg) of < 0.05 were used for further analysis. These differentially expressed genes were then grouped by Pearson correlation and hierarchical clustering. Analysis of silhouette plots identified five distinct clusters of differentially expressed genes for downstream analysis. Clusters of interest were analyzed with the Database for Annotation, Visualization and Integrated Discovery tool (Huang et al., 2009b, Huang et al., 2009a) and visualized by the Enrichment Map plugin from Cytoscape. Gene set enrichment analysis (Subramanian et al., 2005) was also performed with C2: CP collection of the Molecular Signature Database (MSigDB).

#### Single cell RNA-Seq

Per-sample raw counts were normalized and variable genes identified. All other QC metrics were within thresholds set by 10x Genomics. The two expression matrices were integrated using a CCA approach. The integrated data were scaled and the dimensionality reduced using PCA. Cells were clustered using a shared nearest neighbor approach producing 14 clusters. Cells with a feature count > 7,500 and a mitochondrial gene count > 25% were removed. We identified the top 2000 variant genes to use for further analysis. We reduced the dimensionality of the data to the first 30 principle components. We clustered the cells at a resolution of 1.2 and displayed the expression of marker genes on a diffusion map projection (UMAP software). Diffusion map projections were produced to show clustering and gene specific expression. All analyses were carried out using Seurat 3 available from CRAN (Stuart et al., 2019).

#### ChIP-qPCR and qPCR

For ChIP-qPCR, significance was calculated for log-transformed percentage of input values for at least 2 biological replicates using unpaired Student’s T-Test. For qPCR of cDNA, all genes were normalized to the housekeeping gene GUSB.

### Data and Code Availability

All raw sequencing datasets (RNA-seq, ChIP-seq and scRNA-seq) that support the findings of this study have been deposited to GEO repository (hosted by NCBI) under the superseries identifier GEO: GSE127937. Subseries numbers are for each experiment set and data type: RNA-Seq: BMP4 time-course (Figures 2 and 3) = GEO: GSE127936; RNA-Seq: TROPH/ENDO/MESO lineage time-courses (Figure 6) = GEO: GSE127935; ChIP-Seq: SMAD1 ChIP Seq in H1 cells (Figure 2) = GEO: GSE135254; ChIP-Seq: GATA3 ChIP Seq in H1 cells (Figure 5) = GEO: GSE135255; scRNA-Seq: 48 hr BMP4 in wt and GATA3 KO cells (Figure S7) = GEO: GSE135253

## Supplemental Information

Document S1. Figures S1–S7 and Table S1

Document S2. Article plus Supplemental Information

## Footnotes

Supplemental Information can be found online at https://doi.org/10.1016/j.stem.2020.03.005.
