# Supplementary material for: GATA3 Mediates a Fast, Irreversible Commitment to BMP4-Driven Differentiation in Human Embryonic Stem Cells
Source: Cell Stem Cell. 2020 May 7;26(5):693–706.e9. doi: 10.1016/j.stem.2020.03.005 (PMC7487786; doi:10.1016/j.stem.2020.03.005)
Supplement: Document S1. Figures S1–S7 and Table S1 [file mmc1.pdf]

Cell Stem Cell, Volume *26*

Supplemental Information

GATA3 Mediates a Fast, Irreversible Commitment to BMP4-Driven Differentiation in Human Embryonic Stem Cells

Alexandra Gunne-Braden, Adrienne Sullivan, Borzo Gharibi, Rahuman S.M. Sheriff, Alok Maity, Yi-Fang Wang, Amelia Edwards, Ming Jiang, Michael Howell, Robert Goldstone, Roy Wollman, Philip East, and Silvia D.M. Santos

Supplemental Information

GATA3 mediates a fast, irreversible commitment to BMP4-driven differentiation in hESCs

Gunne-Braden et al 2020

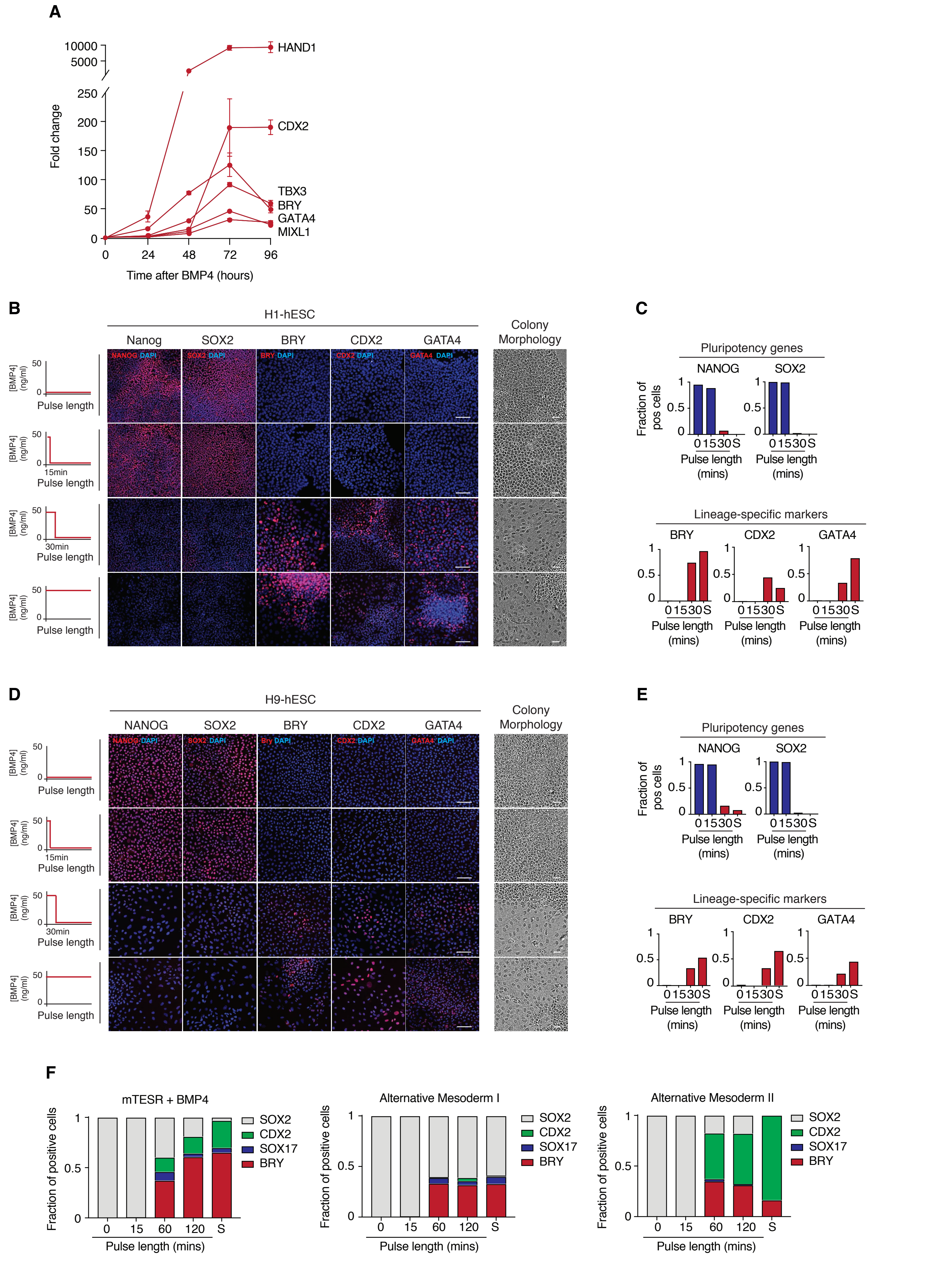

Supplemental Figure 1. Gunne-Braden et al

Supplemental Figure 1. (related to figure 1) hES cell commitment to BMP4-driven differentiation is an early event (**A**) Quantification of canonical (lateral) mesoderm specific gene expression after BMP4 stimulation as measured by qPCR for the indicated times. House-keeping gene GUSB was used for normalisation. Error bars show ± standard deviation (SD). n=3 independent experiments. (**B**) Left: Schematic of duration of BMP4 pulses used to drive differentiation in H1 hES cells. Middle: Representative images of pluripotency markers (NANOG and SOX2) and lineage specific markers (BRY, CDX2 and GATA4) in response to 15 and 30 minutes pulses of BMP4 stimulation (50ng/ml). Cells cultured without BMP4 and with sustained (S) BMP4 were used as controls. Images are shown as the merge image between gene of interest (red channel) and DAPI (blue channel). Scale bar represents 100μm. Right: Representative images of colony morphology for the different experimental conditions. Scale bar represents 50μm. (**C**) Quantification of the fraction of positive cells showing expression of pluripotency (NANOG and SOX2) and mesoderm-specific (BRY, CDX2 and GATA4) genes at day 3 following different pulses of BMP4. n>500 cells were analysed for each experimental condition. (**D**) Left: Schematic of duration of BMP4 pulses used to drive differentiation in H9 hES cells. Middle: Representative images of pluripotency (NANOG and SOX2) and lineage specific markers (BRY, CDX2 and GATA4) in response to 15 and 30 minute pulses of BMP4 stimulation (50ng/ml). Cells cultured without BMP4 and with sustained (S) BMP4 were used as controls. Images are shown as the merge image between the gene of interest (red channel) and DAPI staining (blue channel). Scale bar represents 100μm. Right: Representative images of colony morphology for the different experimental conditions. Scale bar represents 50μm. (**E**) Quantification of the fraction of positive cells showing expression of pluripotency (NANOG and SOX2) and mesoderm specific (BRY, CDX2 and GATA4) genes at day 3 following different pulses of BMP4. n>200 cells were analysed for each experimental condition. (**F**) Quantification of the fraction of positive cells for pluripotency (SOX2) and lineage-specific (CDX2, BRY, SOX17) markers in response to different pulses of mesoderm-inducing stimuli using different protocols for mesoderm induction. Cells cultured without stimulation and with 72 hours of sustained treatment (S) were used as controls. Three different protocols to induce mesoderm differentiation are shown: BMP4 in mTESR (left), alternative mesoderm protocol I (middle) and alternative mesoderm protocol II (right). Please see "Differentiation

of hES cells in chemically defined conditions” for mesoderm differentiation protocols details above. n>200 cells were analysed for each experimental condition.

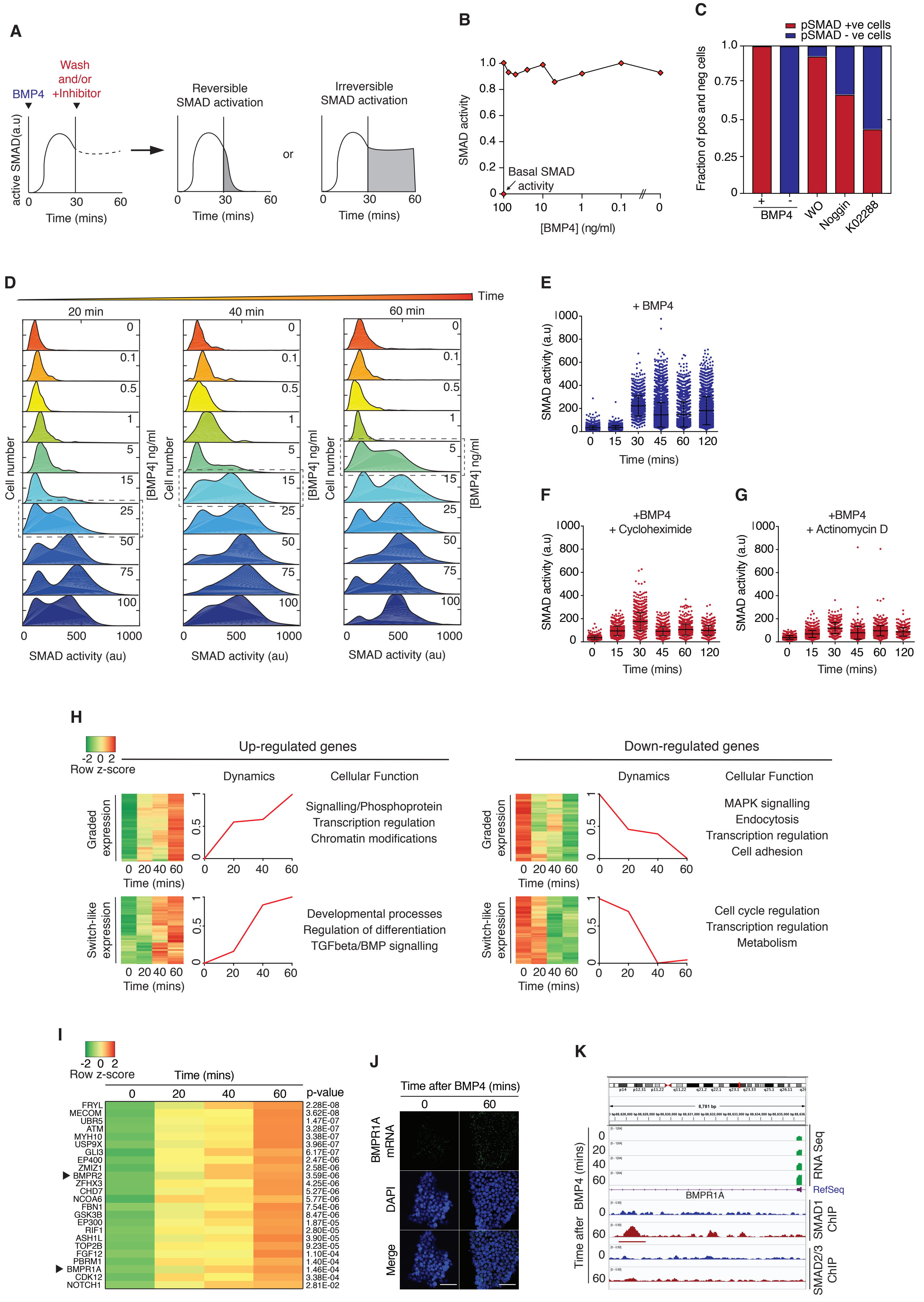

Supplemental Figure 2. Gunne-Braden et al

Supplemental Figure 2. (related to figure 2) Positive feedback promotes switch-like, irreversible activation of the SMAD regulatory network and sustains BMP4 signals (**A**) Schematic showing experimental set up and outcomes to test SMAD irreversibility after BMP4 stimulation. hES cells were stimulated with BMP4 for 30 minutes to allow for maximum SMAD activation. BMP4 was then removed followed by either washing or treatment with BMP4 pathway inhibitors. Reversible SMAD activation would result in SMAD activity returning to basal levels following inhibition or BMP washout, while irreversible activation of SMAD would result in maintenance of SMAD activity following inhibition or BMP washout. (**B**) Quantification of SMAD activation dynamics in response BMP4 concentration at the indicated levels. SMAD activity after 60 minutes of BMP4 (100ng/ml) was set as 1. Basal level of SMAD activation at time 0 minutes is shown. (**C**) Percentage of cells showing active (red) and inactive (blue) SMAD at 60min of BMP4 wash out (WO), stimulation with BMPR inhibitor (K02288) or BMP4 inhibitor (Noggin). Cells treated with BMP4 or left untreated are shown as controls. n>200 cells were analysed for each experimental condition. (**D**) Quantification of SMAD activation dynamics as a function of BMP4 concentration (0-100ng/ml) and BMP4 treatment duration (20-60 minutes). The shorter the pulse of BMP4 stimulation, the higher the BMP4 concentration needed to activate SMAD is highlighted (dash square). n>10^4 cells were analysed for each experimental condition. n=3 independent experiments. (**E**) Quantification of SMAD activation dynamics after BMP4 stimulation. n>200 cells were analysed for each experimental condition. (**F**) Quantification of SMAD activation dynamics after BMP4 stimulation in the presence of translation inhibitor cycloheximide. n>200 cells were analysed for each experimental condition. (**G**) Quantification of SMAD activation dynamics after BMP4 stimulation in the presence of transcription inhibitor actinomycin D. n>200 cells were analysed for each experimental condition. (**H**) Heat-maps showing four different clusters of differentially expressed genes after 0, 20, 40 and 60 minutes of BMP4 treatment. Line plots show mean dynamics of gene clusters, demonstrating upregulation and down-regulation of genes in a graded and switch-like manner. Top GO terms representative of genes within each cluster are shown. (**I**) Heat-map of a subset of RNA-Seq-based gene expression profiles showing graded expression dynamics after BMP4 stimulation. p-values for differential expression of each gene are shown. BMP type I and type II receptors are highlighted. (**J**) Representative

images showing BMP type 1 receptor (BMPR1A) expression using RNA-FISH signal following 60 minutes BMP4 stimulation (50ng/ml). Scale bar represents 100μm. (**K**) SMAD1 ChIP-Seq analysis of intron 2 of BMP receptor (BMPR1A) in the presence (red) or absence (blue) of BMP4 showing positive feedback specifically between SMAD1/5/8 and its activator, BMPR1A. Significant peak region relative to input chromatin is highlighted. Density of aligned reads from RNA-Seq experiments with 0, 20, 40 or 60 minutes treatment with BMP4 (50ng/ml) is shown (green).

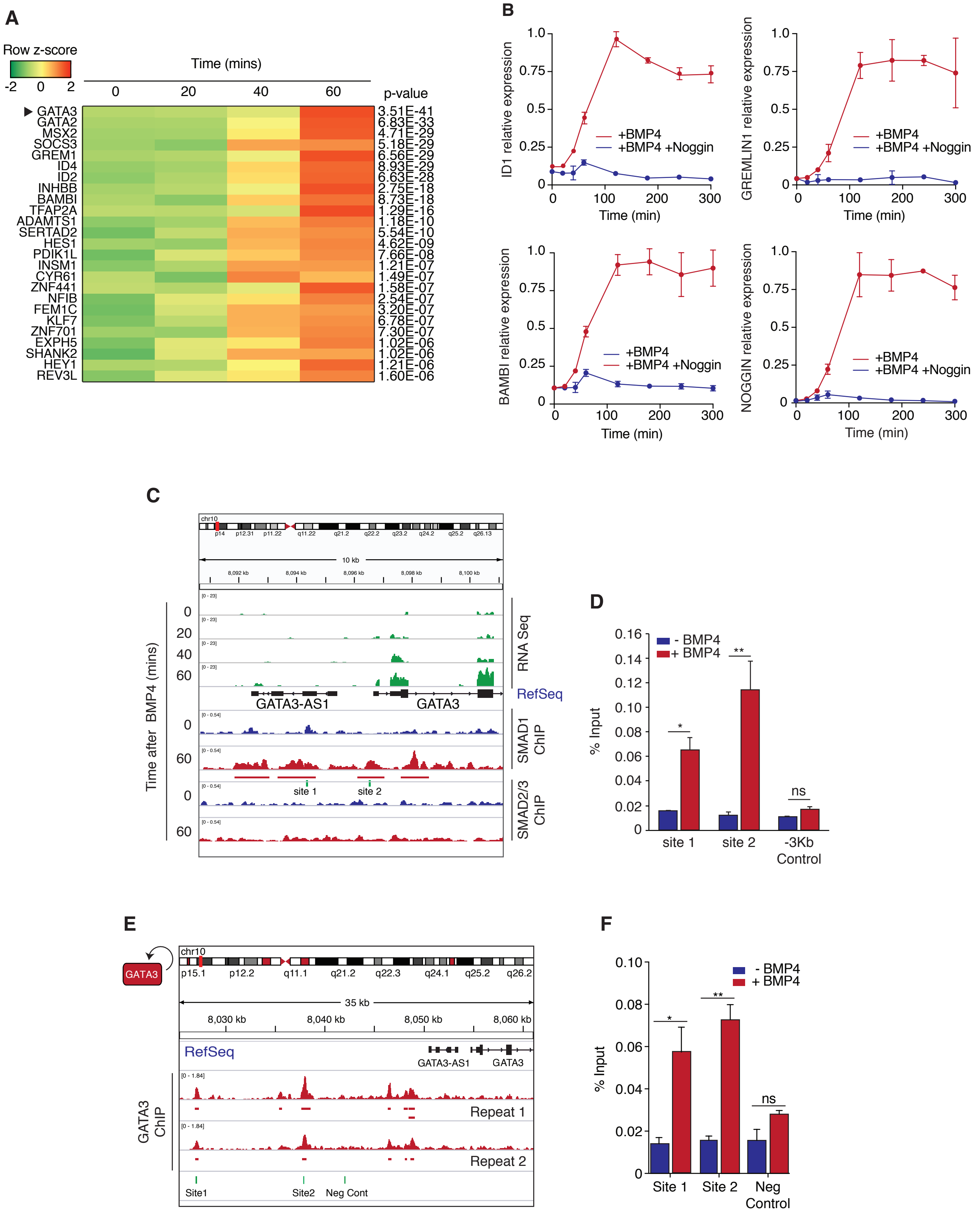

Supplemental Figure 3. Gunne-Braden et al

Supplemental Figure 3. (related to figure 3) GATA3 mirrors SMAD switch-like, irreversible activation dynamics and decodes BMP4 signals (**A**) Heat-map of a subset of RNA-Seq-based gene expression profiles showing switch-like dynamics for differentially expressed genes after BMP4 stimulation. p-values for differential expression of each gene are shown. GATA3 is highlighted. (**B**) Quantification of canonical SMAD target genes after BMP4 stimulation in the presence (red) or absence (blue) of Noggin (100ng/ml), as measured by qPCR. House-keeping gene GUSB was used for normalisation. Error bars show ± standard deviation from n=3 biological replicates. (**C**) SMAD1/5/8 ChIP-Seq analysis of the GATA3 promoter region in the presence (red) or absence (blue) of BMP4 showing specific binding of SMAD1/5/8 to GATA3 promoter. Significant peak regions relative to input chromatin are highlighted. Density of aligned reads from RNA-Seq experiments with 0, 20, 40 or 60 minutes treatment with BMP4 (50ng/ml) is shown (green). (**D**) Quantification of SMAD1 ChIP-qPCR using site 1 and site 2 as amplicons in the presence (red) or absence (blue) of BMP4 (50ng/ml). A primer set outside of significant regions was used as a negative control. Error bars show mean ± SEM for n=3 biological replicates (*p<0.05, **p<0.01, ns is not significant). (**E**) GATA3 ChIP-Seq analysis of its own promoter after BMP4 stimulation showing potential auto-regulation. Significant peak regions relative to input chromatin are highlighted. Amplicons used for ChIP qPCR (site1 and site 2) are shown. n=2 biological replicates. (**F**) Quantification of GATA3 ChIP qPCR using site 1 and site 2 as amplicons in the presence (red) or absence (blue) of BMP4 (50ng/ml). A primer set outside the significant peak regions was used as a negative control (Neg Cont). Error bars show mean ±SEM for n=3 biological replicates (*p<0.05, **p<0.01, ns is not significant).

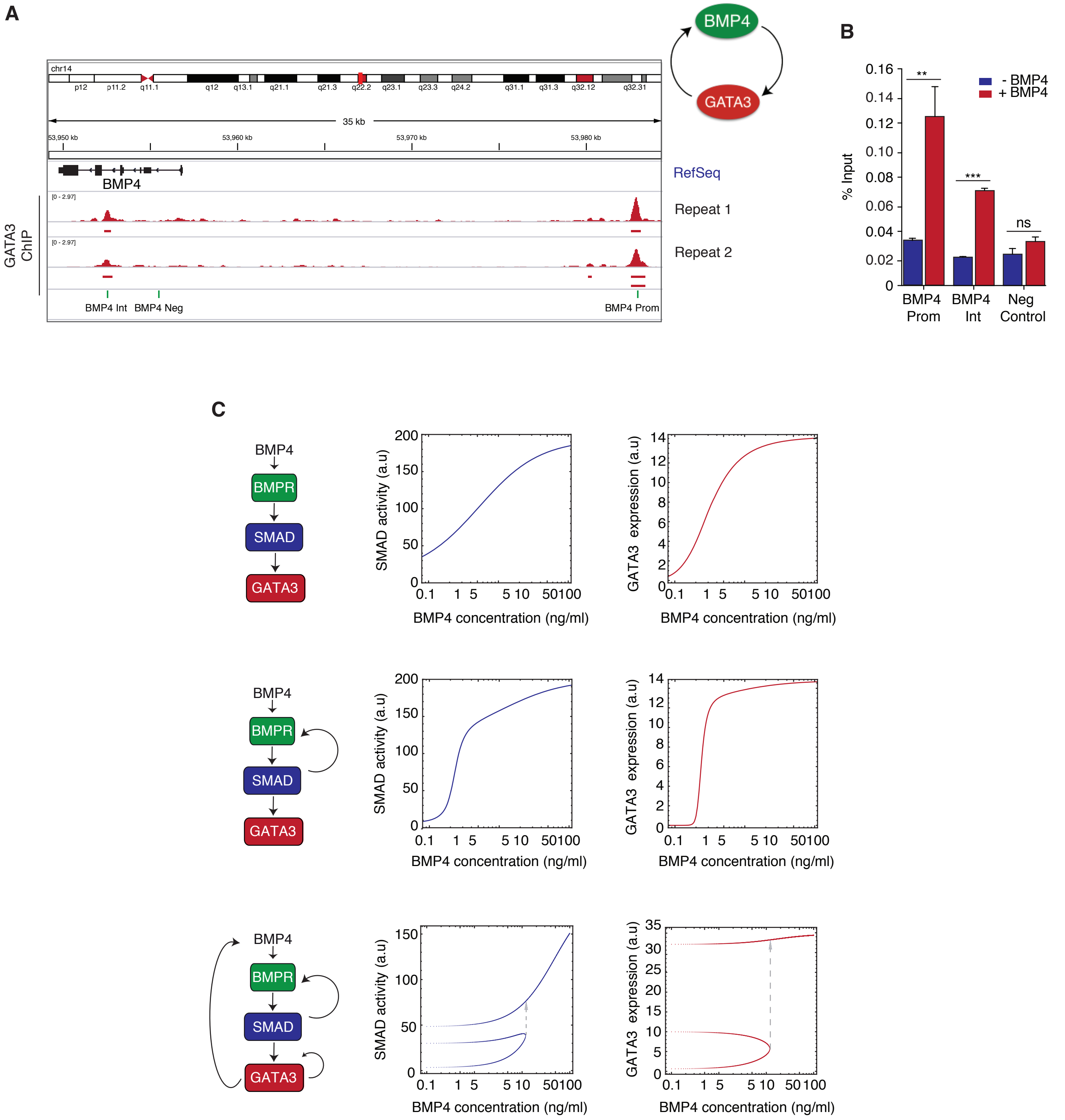

Supplemental Figure 4. Gunne-Braden et al

Supplemental Figure 4. (related to figure 4) Interlinked positive feedbacks give rise to irreversible SMAD activation and GATA3 expression after BMP4 stimulation (**A**) GATA3 ChIP-Seq analysis of BMP4 gene and BMP4 promoter region after BMP4 treatment. Significant peak regions relative to input chromatin are highlighted. Amplicons used for ChIP qPCR (BMP4 Int and BMP4 Prom) are shown. n=2 biological replicates (**B**) Quantification of GATA3 ChIP qPCR using BMP4 Int and BMP4 Prom as amplicons in the presence (red) or absence (blue) of BMP4 (50ng/ml). A primer set outside the significant peak regions was used as a negative control (Neg Control). Error bars show mean ±SEM for n=3 biological replicates (**p<0.01, **p<0.001, ns is not significant). (**C**) (top) Schematic of simplified BMP4 transduction network without feedback regulation used in the ODE model. Model simulation of the hyperbolic steady-state responses of SMAD activation (left) and GATA3 expression (right) as a function of BMP4 concentration in the absence of feedback regulation. (middle) Schematic of simplified BMP4 transduction network with SMAD->BMP receptor->SMAD feedback regulation used in the ODE model. Model simulation of the sigmoidal steady-state responses of SMAD activation (left) and GATA3 expression (right) as a function of BMP4 concentration in the presence of SMAD->BMP receptor->SMAD feedback regulation. (bottom) Schematic of simplified BMP4 transduction network with three interlinked positive feedbacks used in the ODE model. Model simulation of the steady-state responses of SMAD activation (left) and GATA3 expression (right) as a function of BMP4 concentration in the presence of three positive feedbacks shows irreversibility in SMAD activation and GATA3 expression.

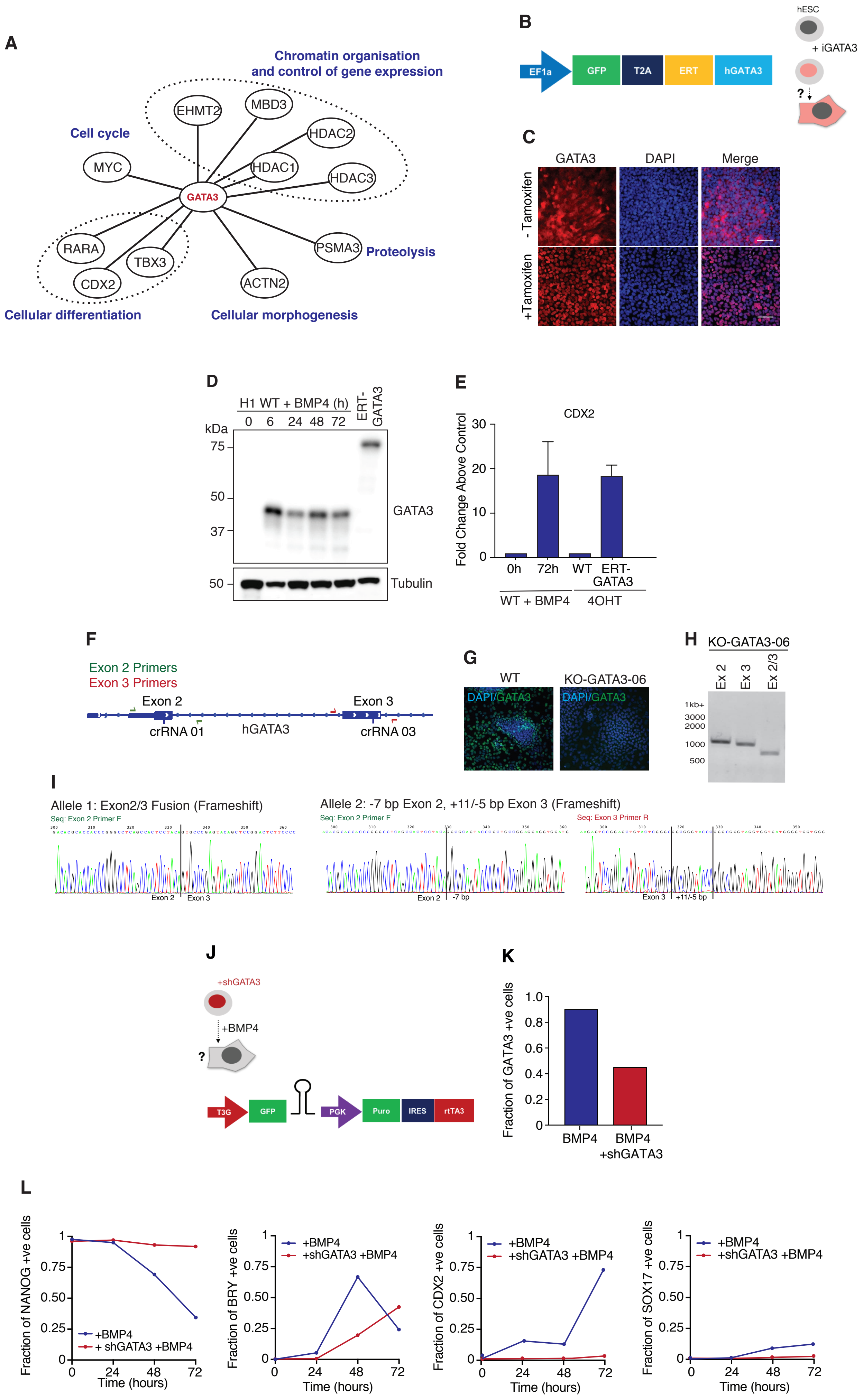

Supplemental Figure 5. Gunne-Braden et al

Supplemental Figure 5. (related to figure 5) GATA3 is necessary for timely differentiation and GATA3 induction in hESC can drive differentiation in the absence of BMP4 signals (**A**) Genes identified by Reactome as potential GATA3 binding partners and their associated cellular functions. (**B**) Schematic of the experimental set up to test whether inducing GATA3 expression in pluripotent hES cells promotes differentiation and of tamoxifen-inducible GATA3 construct used to make stable hES cell lines. (**C**) Representative images showing GATA3 translocation from the cytoplasm to the nucleus of hES cells in the presence of 1μM tamoxifen. Scale bar shows 50μm. (**D**) Western blot analysis testing Tamoxifen (4OHT)-induced ERT-GATA3 and showing that levels of exogenous ERT-GATA3 expressed in H1 hES cells are similar to levels of endogenous GATA3 expressed when H1 hES cells are treated with BMP4 for 6, 24, 48, or 72 hours. Tubulin is used as a loading control. (**E**) qPCR of the known GATA3 target gene CDX2 shows that similar levels are induced when hES cells are differentiated with BMP4 (50 ng/ml), or when ERT-GATA3 activity is induced with 4OHT for 4 days. Histogram shows mean fold change above matched control samples. n=2, error bars show standard deviation. (**F**) Schematic of human GATA3 gene, showing approximate binding sites of CRISPR RNA (crRNA) sequences (crRNA 01, crRNA 03; Dharmacon) and primers for amplification of Exon 2 (green) and Exon 3 (red). (**G**) Immunofluorescent staining shows that after 2 days differentiation with BMP4 (50 ng/ml), H1 WT cells (left panel) show GATA3 expression (green) whereas the derived monoclonal line KO-GATA3-06 (right panel) shows no expression. Nuclei are stained with DAPI (blue). (**H**) PCR of gDNA from monoclonal line KO-GATA3-06 shows expected sizes for amplification of Exon 2 (1068 bp) and Exon 3 (990 bp), but also indicates a large deletion between Exon 2 and Exon 3 (expected size is 3686 bp). (**I**) Sequencing of gDNA from KO-GATA3-06 shows that both alleles have been successfully edited to produce a frameshift in the transcript of GATA3. CRISPR-Cas9 editing has produced a fusion between Exon 2 and Exon 3 in Allele 1. (**J**) Schematic of the experimental set up to test hESC differentiation after BMP4 treatment when GATA3 expression levels have been perturbed and of doxycycline-inducible shRNA construct to establish stable hES cells where GATA3 has been knocked-down. (**K**) Quantification of the fraction of GATA3 expressing cells at 48 hours following BMP4 stimulation (50ng/ml) in the presence (red) or absence (blue) of doxycycline (1μg/ml) to induce shRNA against GATA3. n>500 cells were analysed for each experimental

condition. (**L**) Quantification of the fraction of positive cells for pluripotency gene (NANOG) and lineage specific markers (BRY, SOX17 and CDX2) in response to BMP4 (50ng/ml) stimulation in the presence (red) or absence (blue) of doxycycline (1μg/ml) to induce RNA interference against GATA3 (shGATA3). n>500 cells were analysed for each experimental condition.

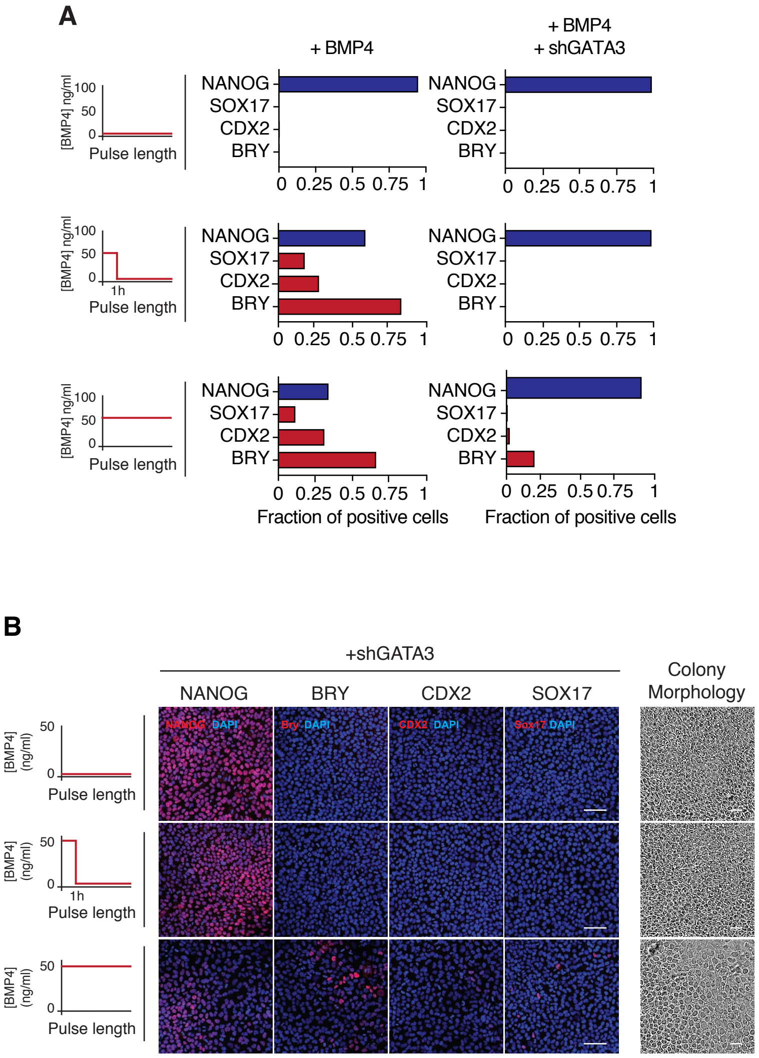

Supplemental Figure 6. Gunne-Braden et al

Supplemental Figure 6. (related to figure 6) GATA3 is an early commitment gene and is required for early commitment to differentiation (**A**) Left: Schematic of duration of BMP4 pulses used to drive hES cells differentiation. Right: Quantification of the fraction of positive cells showing expression of pluripotency genes (NANOG, SOX2 and OCT4) and mesoderm-specific genes (BRY, CDX2 and GATA4) at day 3 following different pulses of BMP4 treatment in the presence or absence of GATA3 knockdown (shGATA3). shGATA3 was induced by doxycycline (1µg/ml). n>500 cells were analysed for each experimental condition. (**B**) Left: Schematic of duration of BMP4 pulses used to drive hESC differentiation. Middle: Representative images of pluripotency (NANOG) and lineage-specific markers (BRY, CDX2 and SOX17) in response to a pulse of BMP4 (50ng/ml) for 60 minutes in hES cells where GATA3 has been knocked-down (shGATA3). Cells cultured with or without BMP4 for the length of the experiment were used as controls. Images are shown as the merge image between the gene of interest (red channel) and DAPI (blue channel). Scale bar represents 100µm. Right: Representative images of colony morphology for the different experimental conditions. Scale bar represents 50µm.

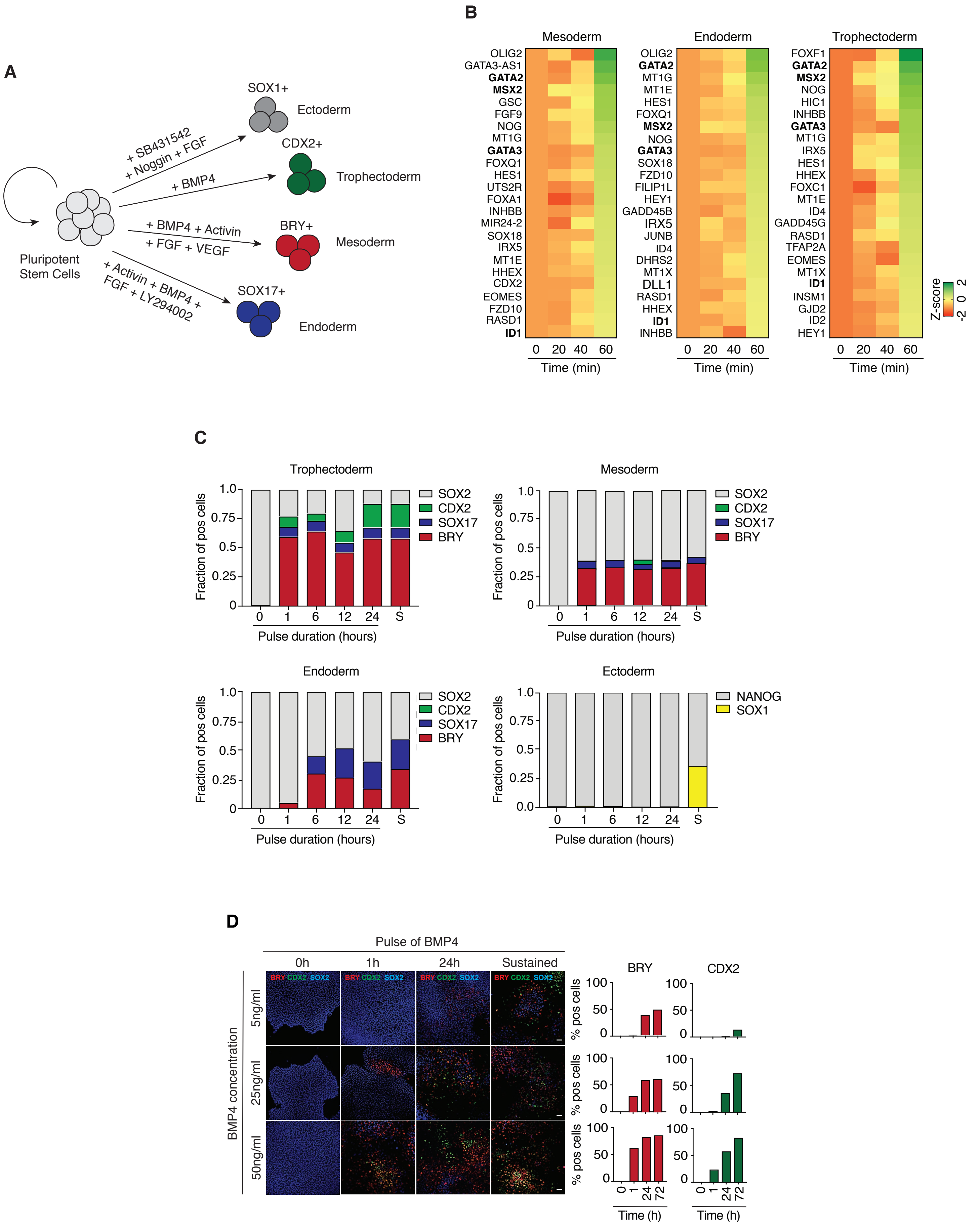

Supplemental Figure 7. Gunne-Braden et al

Supplemental Figure 7. (related to figure 7) Early pluripotency exit is conserved in BMP4 induced fate-choices (**A**) Schematic of different differentiation protocols to induce endoderm, mesoderm, ectoderm and trophectoderm differentiation of hES cells. (**B**) Heat-maps of a subset of RNA-seq-based gene expression profiles for mesoderm, endoderm and trophectoderm fates showing conserved expression of GATA3 and other potential early commitment genes as well as their switch-like dynamics. Shared genes between all fates are highlighted. (**C**) Quantification of the fraction of positive cells expressing pluripotent or lineage-specific markers in response to stimuli to promote endoderm, mesoderm, ectoderm or trophoblast differentiation of hES cells. Untreated cells (0) and cells treated for the duration of the experiment (S) are shown as controls. n>200 cells for each experimental condition. (**D**) Left: Montage showing representative images of Brachyury, CDX2 and SOX2 expression in cells stimulated with high (50ng/ml), medium (25ng/ml) and low (5ng/ml) BMP4 levels. Cells were pulsed with BMP4 for the indicated times. After two washes with PBS, cells were returned to pluripotency growth media (mTESR1) until a total 72h, after which cells were fixed and stained for the indicated markers. Sustained stimulation refers to continuous stimulation for 72h. Right: Percentages of cells expressing differentiation markers Brachyury (BRY) and CDX2 are shown for the experimental conditions described. n>500 cells were analysed per experimental condition. Scale bar shows: 50μm.

Primers for ChIP qPCR		
Name	*Primer 1 (5' - 3')*	*Primer 2 (5' - 3')*
hGATA3 Prom Site1 (SMAD1 ChIP)	TCCAAAATTAGCGGATAGGC	GCCAGGTCTTGAAAATGCTG
hGATA3 Prom Site2 (SMAD1 ChIP)	TTGGGTTGCAGTTTCCTTG	CGCAACTTAAGGAGGTTCTAGTG
hGATA3 Prom -3kb Neg (SMAD1 ChIP)	TTCAGGAAGAGTCCCAAAGC	TTCATGCTTCTCAGGTGTGG
hBMPR1A Int2 (SMAD1 ChIP)	TAGAGAACGGGAAGGAATGG	AGGGCCTGGATTAGCATAAG
hBMPR1A -3.5kb Neg (SMAD1 ChIP)	CGCACTAAAGCACCCAATTC	GCCAGCTGTGGATTTCTTTC
hGATA3 Prom Site1 (GATA3 ChIP)	ACCCAACGGCTTTACACAAG	AATGCCACACTGATCCCAAG
hGATA3 Prom Site2 (GATA3 ChIP)	CGAGAGACAAACAGATGAGCAG	CCTTTGCTCCCTTTAAGCAG
hGATA3 Prom -3kb Neg (GATA3 ChIP)	TTCAGGAAGAGTCCCAAAGC	TTCATGCTTCTCAGGTGTGG
hBMP4 Prom (GATA3 ChIP)	TTCAGTGCCAGCTAAGTTCC	AGGGCCTGGATTAGCATAAG
hBMP4 Int (GATA3 ChIP)	ATGCACGGCCTGAAAGTAAG	GATGGAGGCTATCTGATTTGG
hBMP4 Neg (GATA3 ChIP)	AGAACTTTCATCCCCAGAGC	CGGCATTAGGAGAAGATTCG
hCDX2 Int (GATA3 ChIP)	CGCCATTTGCTCAGTAGTGG	CAACACAGCCGCAAACAATG
hHES1 Prom (GATA3 ChIP)	GGCCTGGCCTAATTATACGG	GACGCAGTTTGGAGTCGTG
hFOXC1 Prom (GATA3 ChIP)	ATCAGACTTTGGGGAGATGG	ATAGGAGCGGCAGATAGCAG
hISL1 Prom (GATA3 ChIP)	TCTGGAGGGCTAATCCAAAC	CCTCTTCGGCTGATTCCAG
hSNAI2 Prom (GATA3 ChIP)	CGGAAGAACTGGAAGATTGC	CGGTGGTTCCAAATGACAG
**Primers for GATA3 KO Screening**		
Name	*Primer 1 (5' - 3')*	*Primer 2 (5' - 3')*
hGATA3 Exon 2	CTGCCATACCCAGTTTTTGG	TCTGGGAAAAGGTCAAGGAG
hGATA3 Exon 3	ATGGGTGAAGGATTCTGTCC	TTCCTATCCCAGCTCATTGG
**Primers for RT-qPCR**		
Name	*Primer 1 (5' - 3')*	*Primer 2 (5' - 3')*
ADM	AGAGCATGAACAACTTCC	TGTCCTTATCTGTGAACTG
APLNR	AGCAGAGAGAAGCCTGGC	TGGATGCTTTGAGGACAGCT
BAMBI	CAAGGAAACAGGTATCAG	CAACATAATAAGCAACACTAA
BMP4	ACGAAGAACATCTGGAGAA	TGCTGCTGAGGTTAAAGA

BMPRIA	GAAGATATGCGTGAGGTT	AACTGCTCGTAGACATTC
BRY	GATCACTTCTTTCCTTTGCATCAAG	TGCTTCCCTGAGACCCAGTT
CD8A1	TTACTGCAACCACAGGAACC	GTTAGACGTATCTCGCCGAAAG
CDH1	ATGAGTGTCCCCCGGTATCT	GGCTGTGGGGTCAGTATCAG
CDH2	GTATCCGGTCCGATCTGCA	TCATATGGTGGAGCTGTGGG
CDX2	CAGGACGAAAGACAAATATC	ATGTAGCGACTGTAGTGA
CMKLR1	TACAACACTTCCATCAGTTACGG	TGGTCACCCTGGCTTCC
TNNT2	GGTCGAACTTCTCTGCCTCC	AGCGGAAAAGTGGGAAGAGG
CYR61	AATGCAGCAAGACCAAGA	GGTATTTCTTCACACTCAAACAT
DES1	ACCTGCTCAACGTGAAGATG	TAGGTCTGGATGGGGAGATTG
DLK1	GACGGGGAGCTCTGTGATAG	GCATTCATAGAGGCCATCGTC
DMRT3	GGCACATCTTTGAACACACC	GCAGAAAACCTCAACGTGTC
DPPA3	TGAAAGAAGACCAACAAACA	CCATAGGTTCTAAAGATACAG
DPPA4	CGCACCTTGAAGACAATA	TTTCCTGATATTCTATTCCCATT
DPPA5	TTTCCTGATATTCTATTCCCATT	ATTGGCTGGAAACTGGTT
EOMES	CGGGGTTGGTATTTGTGTAAGG	ATCATTACGAAACAGGGCAGGC
EPCAM	ATTGTGGTTGTGGTGATAG	TCTCAGCCTTCTCATACTT
FABP7	ACATACAGAAATGGGATGGCA	GAACAGCAACCACATCACCA
FGF4	CCTTCTTCACCGATGAGT	ACTTGTAGGACTCGTAGG
FGF5	ACGAGGAGTTTTCAGCAACA	AAAACGCTCCCTGAACTTGC
FOXA2	CACTCGGCTTCCAGTATG	TGCATAGTAGCTGCTCCA
FOXD3	GGATGGGAGGGAAATTCTTTG	AACTTCGGAGAGTGAACCTT
FOXF1	CAGCCGTATCTGCACCAG	CTCCTTTCGGTCACACATGC
GATA2	CTCCCAGCTCTACTCCAGG	GTGGTGTGAGTCGGGGTG
GATA3	TAACATCGACGGTCAAGGCA	AGGGATCCATGAAGCAGAGG
GATA3 3'UTR	AGCCTGTCCTTTGGACCAC	CCTGCAAAAATGCAAGTCG
GATA4	AAGCAGGACTCTTGGAAC	AGGAATTTGAGGAGGGAAG
GATA6	GGTAATAGCAATAATTCCATTCC	GCTGTAGGTTGTGTTGTG
GBX2	AAGGGAAAGACGAGTCAA	GTAGTCCACATCGCTCTC
GREMLIN1	AATACCTGAAGCGAGACT	TAACAGAAGCGGTTGATG
GSC	AGGGAAGAGGAAGGTAAA	CGAGTTAGGTAAGTAATACG
GZMB1	ACCTGCTCAACGTGAAGATG	TAGGTCTGGATGGGGAGATTG
HAND1	CTACATCGCCTACCTGAT	TTCTTGAGTTCAGCCTTG
HES1	GGCGTTAATACCGAGGTG	TAGGTCATGGCATTGATCTG
HEY1	CCTTGCTATGGACTATCG	CTTCAATGATGCTCAGATAAC
HOPX	TCAACAAGGTCGACAAGCAC	GGCGCTGCTTAAACCATTTC
HOXB7	ATGCGAAGCTCAGGAACTG	CGTCAGGTAGCGATTGTAGTG
HOXC8	CTCATGCCCTGGGACTGAC	TCCCGGCAGTTTATCCTTGT
ID1	ACGACATGAACGGCTGCTACT	TTGCTCACTTTGCGGTTCTG
ID2	CGCATCCCACTATTGTCAGC	CAGTGCTTTGCTGTCATTTGA
ID3	ACCTTCCCATCCAGACAG	CCGAGTCAGTGGCAAAAG
ID4	CGCGGTGAACAAGCAGGG	CCTCCCTCTCTAGTGCTCCT
INHBB	GCCTATACTTCTTCATCTC	CAGGAGTTTCAGGTAAAG
KDR	TCATTGTTCCCAGCATTTCA	TTTTTGCCCTTGTTCTGTCC
KIT	ACATAATGAAGACTTGCT	AATATGATTGGTGCTCTC
KLF2	CAAGACCTACACCAAGAGTTC	TTGCAGTGGTAGGGCTTC
KLF4	ACCTACACAAAGAGTTCCC	CCAGTCACAGTGGTAAGG
LEFTY1	TGGACAAATGCTCTGTGCTC	TCCAGTGGCCAAAGATTCTC

LEFTY2	TGGACAGGGAATTGGGATAC	GAGGAAATGACGAGCCAAAG
LHX1	GCGTCCAGTGCTGTGAATG	TACCGAAACACCGGAAGAAG
MEG3	TCACCTGGATGCCTACGTG	GGAATAGGTGCAGGGTGTCC
MEOX1	CGGCTCCGCAGATATGAGAT	CCTTCACACGCTTCCACTTC
MESP1	CCTCCTGCTTGCCTCAAAGTGTC	TCGAAGTGGTTCCTTGGCAGAC
MIXL1	ATTATCCTCAACCACTGT	AGTTCACATCTACCTCAA
MKL2	CAATGCCAGTGAATACAG	CCATAGGTTCTAAAGATACAG
MSGN1	GATGAGGACCTTGGCAGATG	CTTGAGTGTCTGGATCTTGGTG
MSX1	CTCCTCAAGCTGCCAGAAGA	CTTGTGTTTGCGGAGGGTG
MSX2	AATTCAGAAGATGGAGCGGC	CTTGTGTTTCCTCAGGGTGC
NANOG	CCGAAGAATAGCAATGGT	CTGGTGGTAGGAAGAGTA
NCAM1	GCGTTGGAGAGTCCAAATTC	GTTTGGGGTGAGCTTTTCTC
NDP1	CACCCATTGTACAAGTGTAGCTC	AGTGCTGAACGACACCAAAG
NES	CTCCAAGACTTCCCTCAGCT	TCAGGACTGGGAGCAAAGAT
NKD2	CGGTGAACACATCTGAAG	TTGGAATCAAAGCAGGTTT
NKX2.5	GGCGGGCGACGGCGAGATAGC	CCCTGACCGATCCCACCTCAAC
NODAL	CGGTTCTCATGCTCTACTCCAACCG	GGCTTCTGTCTGGCAAATGATG
NOG	CTTGCGATGCTTTTTGTGAC	CGTGGAGCAGTTTTACAGAC
NOTCH2	GTGAGTATGAAGTGGATGA	AATGGTTCACAAGGTCAA
POU5F1/OCT4	ACACTCGGACCACATCCTTC	AGTGAGAGGCAACCTGGAGA
OSR1	ACATCTGCCACAAAGCCTTC	CCTTTCCCACACTCTTGACAC
OTX1	CTCAAGATCAACCTGCCGGA	CTCTTGGTTCCGCTCCCG
PAX6	TGCAGACACACATGAACAGTC	CTTCCGGGAACTTGAACTGG
PAX3	CCACCTATAGCACCACAGGC	ACGCGATATCTGGCTTGAGA
PDGFRA	GACACGGATGTGAGGTGAGG	GGAGGAGAACAAAGACCGCA
SMAD2	AGAATACCGAAGGCAGAC	TGTCCAACCACTGTAGAG
SMAD7	GAGGAGAAGACGAGAGTG	CCTGAGGTAGATCATAGAAGA
SNAI1	ACCACTATGCCGCGCTCTT	GGTCGTAGGGCTGCTGGAA
SOX1	GCTCCAATTCAAATTAGTG	ATACGATGAGTGTTACCT
SOX2	TGGTTGTCTATTAACTTGT	CTCTCAGTCCTAGTCTTA
SOX7	CCAAGGACGAGAGGAAAC	GACTTTCCCAGCATCTTG
SOX17	TTTCATGGTGTGGGCTAA	CAGCATCTTGCTCAACTC
TAGLN	CATGTTCCAGACTGTTGACCTC	CCATCATTCTTGGTCACTGC
TBX2	CACATGCTGGCATCTCAG	CTGCCATGTAGGTGTAGG
TBX3	GATGAGTCCTCCAGTGAA	GACATAAATCTTTGAGGTTCG
TBX6	CCCTTCCCTCTACCATAC	AGCAGTGGTTCAGTACAT
TDO2	GGTGGTTCCTCAGGCTATCA	TGTCGGGGAATCAGGTATGT
TERT	AAGCATTCCTGCTCAAGC	TCTGGGCTGTCCTGAGTG
TFAP2A	CCCCTGGAGCTGTCAAGTAA	CTCGCAGTCCTCGTACTTGA
TUBB3	GCTCAGGGGCCTTTGGAC	CCCCTCCGTGTAGTGACC
WNT3	AACAAGCACAACAACGAGGC	GTCTTCACCTCACAGCTGC
ZBTB7B	CACATGAGGACCCACACA	CTTGTCGTTCCTGGTGAATC
ZIC3	ATGAATCTCAAGGGTCAG	TGGTATCTTTACTGTTTGC
PAX6	TGCAGACACACATGAACAGTC	CTTCCGGGAACTTGAACTGG
PAX3	CCACCTATAGCACCACAGGC	ACGCGATATCTGGCTTGAGA
PDGFRA	GACACGGATGTGAGGTGAGG	GGAGGAGAACAAAGACCGCA

Supplemental Table 1: List of primers used in this study (related to Star Methods).
